# Supplementary material for: Historical biogeography supports Point Conception as the site of turnover between temperate East Pacific ichthyofaunas
Source: PLoS One. 2023 Sep 19;18(9):e0291776. doi: 10.1371/journal.pone.0291776 (PMC10508600; doi:10.1371/journal.pone.0291776)
Supplement: S1 File — (PDF) [file pone.0291776.s001.pdf]

Supporting Information

Historical biogeography supports Point Conception as the site of turnover between temperate East Pacific ichthyofaunas

Table of Contents:

Figure S1: Biogeographic affinities of offshore deep-sea fishes by latitudinal band

Note S1: Post-hoc analysis of range shifts given the null expectation

Figure S2: Simulated proportion of dispersals from north vs south

Table S1: Range shifts of nearshore California fishes since the 1970s

Table S2: New species of nearshore fishes in California waters since the 1970s

Table S3: Species with unclear range information

Table S4: Region-of-origin inferred for California ray-finned fish species

Table S5: Region-of-origin inferred for California cartilaginous fish species

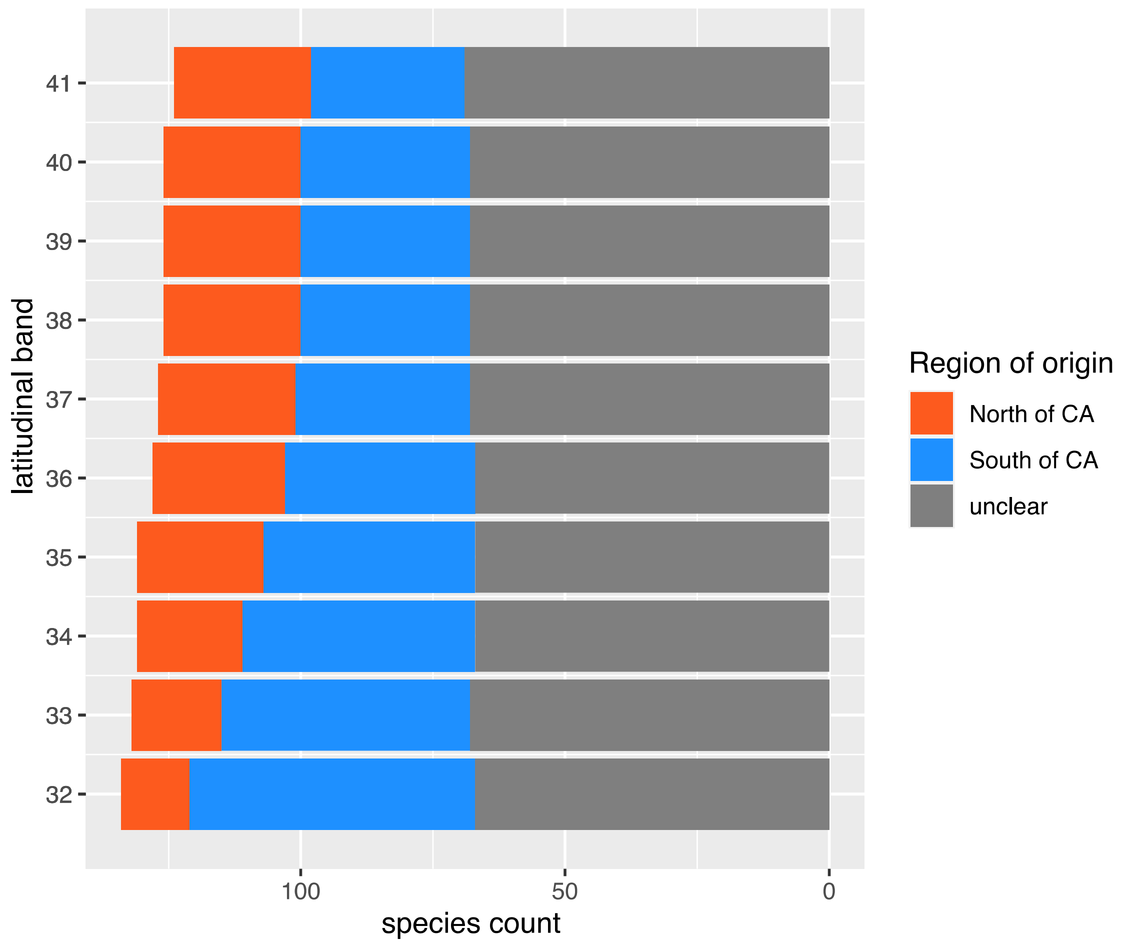

Figure S1: Turnover of northern-and-southern origin species with latitudinal band in California waters. This figure shows results for deep-sea species only; see Tables S4–S5 for a list of species included. All range data for these species were based on modern sources (Love and Passarelli 2020; Love et al. 2021).

Note S1: Post-hoc analyses of range shifts given the null expectation

In this study I report that most new species in California waters since the 1970s dispersed from Baja California, Mexico (northward dispersal, n=69 species) as opposed to dispersal from Oregon (southward dispersal, n=15 species; Table S2) (excluding new species added from recent species descriptions). This is important because dispersals northward are consistent with an effect of anthropogenic climate change on range shifts. However, this is complicated by the fact that species richness is higher towards the equator (i.e. the latitudinal diversity gradient). Since there are more species in Baja California than in Oregon, the pool of species that can potentially disperse into California is also greater. Therefore, we would expect more species to disperse northward under a null model if dispersal was random.

I simulated potential dispersals to test the hypothesis that the number of northward dispersals since the 1970s was even greater than expected. I determined the pool of species that could have potentially dispersed from Oregon to California since 1978 to be 293 species. This is the number of species known to be restricted to North of California today, plus the number reported to have dispersed to California in this study (these species would have been restricted to North of California in 1978). The same logic was applied to determine the pool of species that could have potentially dispersed from Baja California to California to be 728 species in 1978. I performed trials where I randomly sampled 84 species (the observed number) from the total pool of potential dispersers to disperse to California. I then calculated a ratio of dispersers from the southern pool to dispersers from the northern pool. I performed 100,000 trials to determine a null distribution of ratios.

The median ratio in the simulated distribution is 2.5 dispersal events from Mexico for every 1 dispersal from Oregon. The observed ratio of 4.6 (69 divided by 15 species) is outside of the 95% quantile of the simulated distribution (Figure S2). These results suggest the observed number of species entering California from Mexico is significantly greater than expected at random (given the latitudinal diversity gradient), supporting the assertion that recent dispersals are influenced by anthropogenic climate warming.

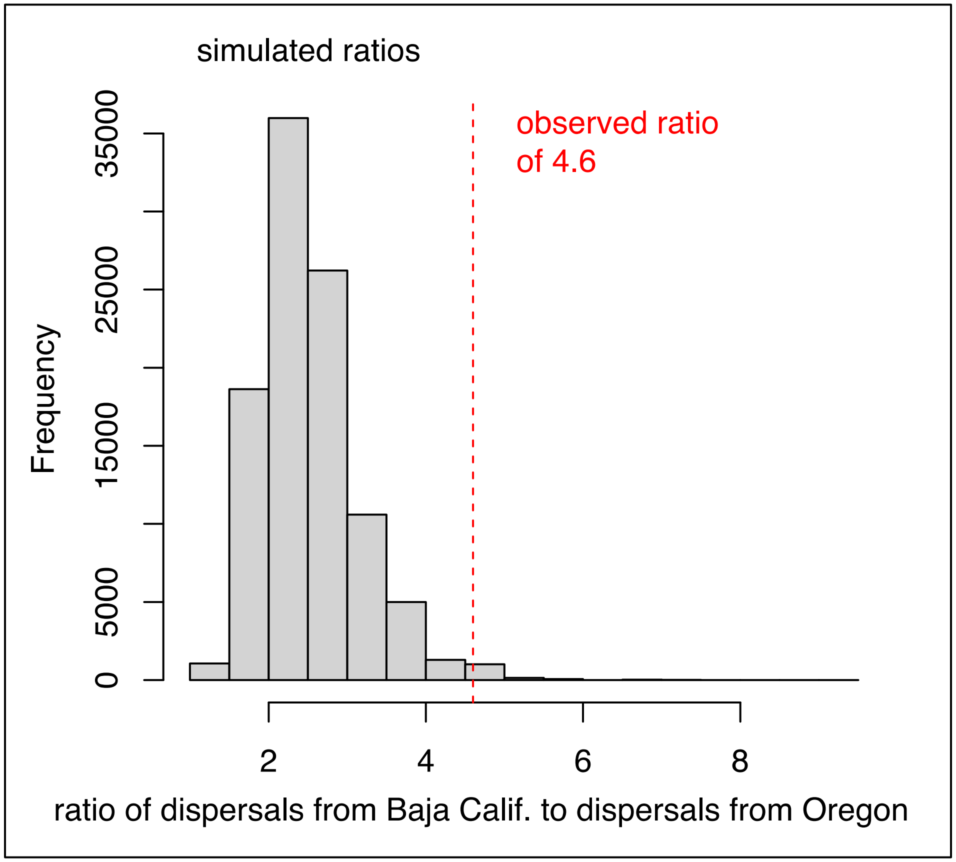

Figure S2. Simulated ratios of species dispersing into California from Baja California (Mexico) versus species dispersing from Oregon (USA). The observed ratio is shown by the red dashed line.

Table S1. List of range changes among inshore marine species between 1978 dataset (Horn and Allen 1978) and this study.

**Class**	**Species (other names in databases)**	**Family**	**1970s N limit**	**New N limit**	**1970s S limit**	**New S limit**	**Type of change**	**Sampled in phylogeny**
Actinopterygii	*Artedius corallinus*	Cottidae	N of CA	41	S of CA	-	N limit constricted southward	yes
Actinopterygii	*Clinocottus recalvus*	Cottidae	42	40	S of CA	-	N limit constricted southward	yes
Actinopterygii	*Lepidocybium flavobrunneum*	Gempylidae	N of CA	33	S of CA	-	N limit constricted southward	yes
Actinopterygii	*Tetrapturus angustirostris*	Istiophoridae	42	40	S of CA	-	N limit constricted southward	yes
Actinopterygii	*Semicossyphus pulcher*	Labridae	37	36	S of CA	-	N limit constricted southward	yes
Actinopterygii	*Sebastes umbrosus*	Sebastidae	37	36	S of CA	-	N limit constricted southward	yes
Actinopterygii	*Epinephelus analogus*	Serranidae	34	33	S of CA	-	N limit constricted southward	yes
Actinopterygii	*Kathetostoma averruncus*	Uranoscopidae	39	36	S of CA	-	N limit constricted southward	yes
Actinopterygii	*Rathbunella hypoplecta*	Bathymasteridae	N of CA	34	S of CA	-	N limit constricted southward (earlier northern records were erroneous)	yes
Chondrichthyes	*Dasyatis dipterura (Hypanus dipterurus)*	Dasyatidae	N of CA	34	S of CA	-	N limit contracted southward (potentially erroneous in 1970s dataset)	yes
Actinopterygii	*Blepsias cirrhosus*	Agonidae	41	N of CA	35	-	N limit extended northward	yes
Actinopterygii	*Leuresthes tenuis*	Atherinopsidae	37	38	S of CA	-	N limit extended northward	yes
Actinopterygii	*Balistes polylepis*	Balistidae	41	N of CA	S of CA	-	N limit extended northward	yes
Actinopterygii	*Hypsoblennius gilberti*	Blenniidae	34	37	S of CA	-	N limit extended northward	yes

Actinopterygii	*Hypsoblennius jenkinsi*	Blenniidae	34	36	S of CA	-	N limit extended northward	yes
Actinopterygii	*Pteraclis aesticola*	Bramidae	35	37	S of CA	-	N limit extended northward	no
Actinopterygii	*Taractichthys steindachneri*	Bramidae	33	34	S of CA	-	N limit extended northward	yes
Actinopterygii	*Caranx caninus*	Carangidae	32	33	S of CA	-	N limit extended northward	yes
Actinopterygii	*Naucrates ductor*	Carangidae	36	N of CA	S of CA	-	N limit extended northward	yes
Actinopterygii	*Trachinotus rhodopus*	Carangidae	34	35	S of CA	-	N limit extended northward	yes
Actinopterygii	*Cebidichthys violaceus*	Cebidichthyidae	41	N of CA	S of CA	-	N limit extended northward	yes
Actinopterygii	*Neoclinus blanchardi*	Chaenopsidae	37	38	S of CA	-	N limit extended northward	yes
Actinopterygii	*Neoclinus stephensae*	Chaenopsidae	36	37	S of CA	-	N limit extended northward	no
Actinopterygii	*Gibbonsia elegans*	Clinidae	35	37	S of CA	-	N limit extended northward	yes
Actinopterygii	*Clinocottus analis*	Cottidae	39	40	S of CA	-	N limit extended northward	yes
Actinopterygii	*Enophrys taurus (Enophrys taurina)*	Cottidae	37	39	33	-	N limit extended northward	yes
Actinopterygii	*Orthonopias triacis*	Cottidae	37	N of CA	S of CA	-	N limit extended northward	yes
Actinopterygii	*Radulinus vinculus*	Cottidae	34	35	34	-	N limit extended northward	no
Actinopterygii	*Symphurus atricauda*	Cynoglossidae	41	N of CA	S of CA	-	N limit extended northward	yes
Actinopterygii	*Diodon hystrix*	Diodontidae	32	34	S of CA	-	N limit extended northward	yes
Actinopterygii	*Remora remora*	Echeneidae	37	N of CA	S of CA	-	N limit extended northward	yes
Actinopterygii	*Phanerodon atripes*	Embiotocidae	38	N of CA	S of CA	-	N limit extended northward	yes
Actinopterygii	*Zalembius rosaceus*	Embiotocidae	38	40	S of CA	-	N limit extended northward	yes

Actinopterygii	*Cheilopogon heterurus (Cypselurus heterurus)*	Exocoetidae	32	33	S of CA	-	N limit extended northward	no
Actinopterygii	*Ruvettus pretiosus*	Gempylidae	36	38	S of CA	-	N limit extended northward	yes
Actinopterygii	*Eucinostomus dowii*	Gerreidae	32	33	S of CA	-	N limit extended northward	no
Actinopterygii	*Girella nigricans*	Girellidae	37	N of CA	S of CA	-	N limit extended northward	yes
Actinopterygii	*Rimicola eigenmanni*	Gobiesocidae	33	34	S of CA	-	N limit extended northward	no
Actinopterygii	*Ctenogobius sagittula (Gobionellus longicaudus)*	Gobiidae	32	33	S of CA	-	N limit extended northward	yes
Actinopterygii	*Anisotremus davidsonii*	Haemulidae	34	36	S of CA	-	N limit extended northward	yes
Actinopterygii	*Xenistius californiensis (Haemulon californiensis)*	Haemulidae	36	37	S of CA	-	N limit extended northward	yes
Actinopterygii	*Istiophorus platypterus*	Istiophoridae	32	33	S of CA	-	N limit extended northward	yes
Actinopterygii	*Tetrapturus audax (Kajikia audax)*	Istiophoridae	34	N of CA	S of CA	-	N limit extended northward	yes
Actinopterygii	*Hermosilla azurea (Kyphosus azureus)*	Kyphosidae	36	41	S of CA	-	N limit extended northward	yes
Actinopterygii	*Halichoeres semicinctus*	Labridae	34	36	S of CA	-	N limit extended northward	yes
Actinopterygii	*Nezumia stelgidolepis*	Macrouridae	41	N of CA	S of CA	-	N limit extended northward	no
Actinopterygii	*Physiculus rastrelliger*	Moridae	40	N of CA	S of CA	-	N limit extended northward	yes
Actinopterygii	*Mugil cephalus*	Mugilidae	36	40	S of CA	-	N limit extended northward	yes
Actinopterygii	*Zalieutes elater*	Ogcocephalidae	34	N of CA	S of CA	-	N limit extended northward	yes
Actinopterygii	*Ophichthus triserialis*	Ophichthidae	40	N of CA	S of CA	-	N limit extended northward	yes
Actinopterygii	*Lactoria diaphana (Ostracion diaphanum)*	Ostraciidae	34	35	S of CA	-	N limit extended northward	yes
Actinopterygii	*Hippoglossina stomata*	Paralichthyidae	36	37	S of CA	-	N limit extended northward	yes

Actinopterygii	*Pleuronichthys verticalis*	Pleuronectidae	37	N of CA	S of CA	-	N limit extended northward	yes
Actinopterygii	*Pristigenys serrula*	Priacanthidae	34	N of CA	S of CA	-	N limit extended northward	yes
Actinopterygii	*Menticirrhus undulatus*	Sciaenidae	34	35	S of CA	-	N limit extended northward	yes
Actinopterygii	*Auxis rochei*	Scombridae	33	37	S of CA	-	N limit extended northward	yes
Actinopterygii	*Euthynnus affinis*	Scombridae	33	35	S of CA	-	N limit extended northward	yes
Actinopterygii	*Thunnus albacares*	Scombridae	35	N of CA	S of CA	-	N limit extended northward	yes
Actinopterygii	*Medialuna californiensis (Medialuna californica)*	Scorpididae	41	N of CA	S of CA	-	N limit extended northward	yes
Actinopterygii	*Sebastes atrovirens*	Sebastidae	38	39	S of CA	-	N limit extended northward	yes
Actinopterygii	*Sebastes chrysomelas*	Sebastidae	40	N of CA	S of CA	-	N limit extended northward	yes
Actinopterygii	*Sebastes ensifer*	Sebastidae	37	N of CA	S of CA	-	N limit extended northward	yes
Actinopterygii	*Sebastes eos*	Sebastidae	37	N of CA	S of CA	-	N limit extended northward	yes
Actinopterygii	*Sebastes gilli*	Sebastidae	36	N of CA	30	-	N limit extended northward	yes
Actinopterygii	*Sebastes hopkinsi*	Sebastidae	37	N of CA	S of CA	-	N limit extended northward	yes
Actinopterygii	*Sebastes levis*	Sebastidae	42	N of CA (northern OR)	S of CA	-	N limit extended northward	no
Actinopterygii	*Sebastes ovalis*	Sebastidae	42	N of CA (WA state)	S of CA	-	N limit extended northward	yes
Actinopterygii	*Sebastes rosenblatti*	Sebastidae	37	40	S of CA	-	N limit extended northward	yes
Actinopterygii	*Sebastes rufus*	Sebastidae	42	N of CA (Canada)	S of CA	-	N limit extended northward	yes
Actinopterygii	*Sebastes semicinctus*	Sebastidae	37	N of CA	S of CA	-	N limit extended northward	yes

Actinopterygii	*Sebastes serranoides*	Sebastidae	41	N of CA	S of CA	-	N limit extended northward	yes
Actinopterygii	*Epinephelus niphobles (Hyporthodus niphobles)*	Serranidae	35	36	S of CA	-	N limit extended northward	yes
Actinopterygii	*Cosmocampus arctus (Bryx arctos)*	Syngnathidae	38	40	S of CA	-	N limit extended northward	no
Actinopterygii	*Syngnathus californiensis*	Syngnathidae	37	38	S of CA	-	N limit extended northward	yes
Actinopterygii	*Synodus lucioceps*	Synodontidae	38	N of CA	S of CA	-	N limit extended northward	yes
Actinopterygii	*Sphoeroides annulatus*	Tetraodontidae	32	33	S of CA	-	N limit extended northward	yes
Actinopterygii	*Desmodema lorum (Desmodena polysticta)*	Trachipteridae	36	N of CA	S of CA	-	N limit extended northward	no
Actinopterygii	*Trachipterus fukuzakii*	Trachipteridae	33	37	S of CA	-	N limit extended northward	no
Actinopterygii	*Zu cristatus*	Trachipteridae	33	34	S of CA	-	N limit extended northward	yes
Actinopterygii	*Lepidopus fitchi*	Trichiuridae	40	N of CA	S of CA	-	N limit extended northward	no
Actinopterygii	*Trichiurus nitens (Trichiurus lepturus)*	Trichiuridae	33	37	S of CA	-	N limit extended northward	yes
Actinopterygii	*Bellator xenisma*	Triglidae	33	34	S of CA	-	N limit extended northward	yes
Actinopterygii	*Lyconema barbatum*	Zoarcidae	42	N of CA (northern Oregon)	S of CA	-	N limit extended northward	no
Agnatha	*Myxine circifrons*	Myxinidae	33	37	S of CA	-	N limit extended northward	NA
Chondrichthyes	*Carcharhinus brachyurus (Carcharhinus remotus)*	Carcharhinidae	33	34	S of CA	-	N limit extended northward	yes
Chondrichthyes	*Carcharhinus leucas*	Carcharhinidae	33	34	S of CA	-	N limit extended northward	yes
Chondrichthyes	*Echinorhinus cookei*	Echinorhinidae	36	N of CA	S of CA	-	N limit extended northward	yes
Chondrichthyes	*Heterodontus francisci*	Heterodontidae	36	37	S of CA	-	N limit extended northward	yes
Chondrichthyes	*Manta birostris (Mobula birostris, Manta hamiltoni)*	Mobulidae	33	34	S of CA	-	N limit extended northward	yes

Chondrichthyes	*Parmaturus xaniurus*	Pentanchidae	36	N of CA	S of CA	-	N limit extended northward	yes
Chondrichthyes	*Platyrhinoides triseriata*	Platyrhinidae	37	38	S of CA	-	N limit extended northward	yes
Chondrichthyes	*Rhincodon typus*	Rhincodontidae	32	41	S of CA	-	N limit extended northward	yes
Chondrichthyes	*Mustelus henlei*	Triakidae	40	N of CA	S of CA	-	N limit extended northward	yes
Chondrichthyes	*Zapteryx exasperata*	Trygonorrhinidae	33	34	S of CA	-	N limit extended northward	yes
Actinopterygii	*Artedius fenestralis*	Cottidae	N of CA	-	34	37	S limit constricted northward	yes
Actinopterygii	*Pseudopentaceros wheeleri (Pentaceros wheeleri)*	Pentacerotidae	N of CA	-	S of CA	35	S limit constricted northward	yes
Actinopterygii	*Oncorhynchus clarkii*	Salmonidae	N of CA	-	37	40	S limit constricted northward	yes
Actinopterygii	*Oncorhynchus nerka*	Salmonidae	N of CA	-	33	34	S limit constricted northward	yes
Actinopterygii	*Allosmerus elongatus*	Osmeridae	N of CA	-	33	34	S limit contracted northward	yes
Actinopterygii	*Agonus acipenserinus (Podothecus accipenserinus)*	Agonidae	N of CA	-	40	38	S limit extended southward	yes
Actinopterygii	*Asterotheca infraspinata (Bathyagonus infraspinatus)*	Agonidae	N of CA	-	40	38	S limit extended southward	yes
Actinopterygii	*Bathyagonus nigripinnis*	Agonidae	N of CA	-	40	33	S limit extended southward	yes
Actinopterygii	*Bothragonus swanii*	Agonidae	N of CA	-	35	33	S limit extended southward	no
Actinopterygii	*Occella verrucosa (Chesnonia verrucosa)*	Agonidae	N of CA	-	37	34	S limit extended southward	no
Actinopterygii	*Ronquilus jordani*	Bathymasteridae	N of CA	-	36	32	S limit extended southward	yes
Actinopterygii	*Clinocottus acuticeps*	Cottidae	N of CA	-	36	33	S limit extended southward	yes
Actinopterygii	*Enophrys bison*	Cottidae	N of CA	-	36	33	S limit extended southward	yes

Actinopterygii	*Jordania zonope*	Cottidae	N of CA	-	34	33	S limit extended southward	yes
Actinopterygii	*Oligocottus rimensis*	Cottidae	N of CA	-	33	S of CA	S limit extended southward	yes
Actinopterygii	*Oligocottus rubellio*	Cottidae	39	-	33	S of CA	S limit extended southward	yes
Actinopterygii	*Radulinus boleoides*	Cottidae	N of CA	-	33	32 (Tanner Bank)	S limit extended southward	yes
Actinopterygii	*Amphistichus rhodoterus*	Embiotocidae	N of CA	-	36	S of CA	S limit extended southward	yes
Actinopterygii	*Gadus macrocephalus*	Gadidae	N of CA	-	34	33	S limit extended southward	yes
Actinopterygii	*Pleurogrammus monopterygius*	Hexagrammidae	N of CA	-	35	33	S limit extended southward	yes
Actinopterygii	*Liparis florae*	Liparidae	N of CA	-	34	33	S limit extended southward	yes
Actinopterygii	*Lumpenus sagitta*	Lumpenidae	N of CA	-	40	36	S limit extended southward	yes
Actinopterygii	*Spirinchus thaleichthys*	Osmeridae	N of CA	-	37	33	S limit extended southward	yes
Actinopterygii	*Thaleichthys pacificus*	Osmeridae	N of CA	-	38	34	S limit extended southward	yes
Actinopterygii	*Apodichthys flavidus*	Pholidae	N of CA	-	33	32	S limit extended southward	yes
Actinopterygii	*Pholis clemensi*	Pholidae	N of CA	-	38	36	S limit extended southward	yes
Actinopterygii	*Pholis ornata*	Pholidae	N of CA	-	36	32	S limit extended southward	yes
Actinopterygii	*Atheresthes stomias*	Pleuronectidae	N of CA	-	33	S of CA	S limit extended southward	yes
Actinopterygii	*Hippoglossoides elassodon*	Pleuronectidae	N of CA	-	37	32	S limit extended southward	yes
Actinopterygii	*Hippoglossus stenolepis*	Pleuronectidae	N of CA	-	33	S of CA	S limit extended southward	yes
Actinopterygii	*Isopsetta isolepis*	Pleuronectidae	N of CA	-	34	33	S limit extended southward	yes
Actinopterygii	*Platichthys stellatus*	Pleuronectidae	N of CA	-	34	33	S limit extended southward	yes

Actinopterygii	*Psettichthys melanostictus*	Pleuronectidae	N of CA	-	33	32	S limit extended southward	yes
Actinopterygii	*Rhamphocottus richardsonii*	Rhamphocottidae	N of CA	-	33	32	S limit extended southward	yes
Actinopterygii	*Sebastes borealis*	Sebastidae	N of CA	-	36	34	S limit extended southward	yes
Actinopterygii	*Sebastes brevispinis*	Sebastidae	N of CA	-	33	S of CA	S limit extended southward	yes
Actinopterygii	*Sebastes caurinus (Sebastes vexillaris)*	Sebastidae	N of CA	-	33	S of CA	S limit extended southward	yes
Actinopterygii	*Sebastes melanops*	Sebastidae	N of CA	-	33	S of CA	S limit extended southward	yes
Actinopterygii	*Sebastes nebulosus*	Sebastidae	N of CA	-	34	33	S limit extended southward	yes
Actinopterygii	*Sebastes nigrocinctus*	Sebastidae	N of CA	-	35	32	S limit extended southward	yes
Actinopterygii	*Anoplarchus purpurescens*	Stichaeidae	N of CA	-	34	33	S limit extended southward	yes
Actinopterygii	*Plagiogrammus hopkinsii*	Stichaeidae	36	-	34	33	S limit extended southward	no
Actinopterygii	*Zaprora silenus*	Zaproridae	N of CA	-	36	34	S limit extended southward	yes
Actinopterygii	*Lycodapus mandibularis*	Zoarcidae	N of CA	-	34	32	S limit extended southward	yes
Chondrichthyes	*Somniosus pacificus*	Somniosidae	N of CA	-	33	S of CA	S limit extended southward	yes
Chondrichthyes	*Lamna ditropis*	Lamnidae	N of CA	-	32	S of CA	S limit extended southward (central Baja)	yes
Chondrichthyes	*Raja stellulata (Beringraja stellulata)*	Rajidae	N of CA	-	32	S of CA	S limit extended southward (central Baja)	yes
Chondrichthyes	*Bathyraja abyssicola*	Arhynchobatidae	N of CA	-	32	S of CA	S limit extended southward (Galapagos Is)	yes
Actinopterygii	*Lophotus capellei (Lophotus cristatus)*	Lophotidae	32	38	S of CA	32 (San Diego)	Both limits shifted northward (range in 1970s dataset may be erroneous)	yes
Actinopterygii	*Eucyclogobius newberryi*	Gobiidae	N of CA	41	32	33	Both N and S limits constricted	yes

Actinopterygii	*Icelinus fimbriatus*	Cottidae	36	N of CA	33	32	Both N and S limits extended	yes
Actinopterygii	*Sebastes lentiginosus*	Sebastidae	33	34	32	S of CA (southern Baja)	Both N and S limits extended	yes
Actinopterygii	*Sebastes phillipsi*	Sebastidae	37	41	33	32	Both N and S limits extended	yes
Actinopterygii	*Hippocampus ingens*	Syngnathidae	33	34	S of CA	32 (San Diego)	Both N and S limits shifted northward	yes
Chondrichthyes	*Alopias superciliosus*	Alopiidae	N of CA	40	33	S of CA	Both N and S limits shifted southward	yes
Chondrichthyes	*Euprotomicrus bispinatus*	Dalatiidae	40	35	33	S of CA	Both N and S limits shifted southward	yes
Chondrichthyes	*Chlamydoselachus anguineus*	Chlamydoselachidae	N of CA	34	34	S of CA	Range from 70s dataset seems erroneous	yes

Table S2. List of new inshore marine species added to California waters since 1978 dataset (Horn and Allen 1978). Ref. [1] = Love and Passarelli 2020. Ref. [2] = Love et al. 2021. Ref. [3] = Horn et al. 2006. Species with a (*) were newly described or revised since the 1978 study, so their addition cannot be attributed to dispersal (see Love and Passarelli 2020 and references therein).

Class	Species (other names in databases)	Family	In 2006 dataset	N limit	S limit	Sampled in phylogeny	Notes
Actinopterygii	*Acanthurus xanthopterus*	Acanthuridae	no	33	S of CA	yes	
Actinopterygii	*Agonomalus mozinoi**	Agonidae	no	N of CA	35	no	
Actinopterygii	*Anoplagonus inermis*	Agonidae	no	N of CA	38	yes	
Actinopterygii	*Bathyagonus alascanus*	Agonidae	no	N of CA	40	yes	
Actinopterygii	*Hemitripterus bolini*	Agonidae	no	N of CA	40	no	
Actinopterygii	*Pallasina aix (Pallasina barbata)*	Agonidae	no	N of CA	37	no	
Actinopterygii	*Apogon atricaudus*	Apogonidae	no	33	S of CA	no	
Actinopterygii	*Apogon retrosella*	Apogonidae	no	32	S of CA	no	
Actinopterygii	*Rathbunella alleni*	Bathymasteridae	no	N of CA	S of CA	no	
Actinopterygii	*Ophioblennius steindachneri*	Blenniidae	no	33	S of CA	yes	
Actinopterygii	*Selar crumenophthalmus*	Carangidae	no	33	S of CA	yes	
Actinopterygii	*Chanos chanos*	Chanidae	no	33	S of CA	yes	
Actinopterygii	*Opisthonema libertate*	Clupeidae	no	34	S of CA	yes	
Actinopterygii	*Opisthonema medirastre*	Clupeidae	no	33	S of CA	yes	
Actinopterygii	*Coryphaena equiselis*	Coryphaenidae	no	32	S of CA	yes	
Actinopterygii	*Asemichthys taylori*	Cottidae	no	N of CA	36	no	
Actinopterygii	*Cottus asper*	Cottidae	no	N of CA	34	yes	
Actinopterygii	*Icelinus limbaughi**	Cottidae	no	34	32	no	
Actinopterygii	*Symphurus oligomerus**	Cynoglossidae	no	34	S of CA	no	
Actinopterygii	*Diodon eydouxii*	Diodontidae	no	33	S of CA	no	
Actinopterygii	*Fistularia commersonii*	Fistulariidae	no	33	S of CA	yes	
Actinopterygii	*Bollmannia stigmatura*	Gobiidae	no	33	S of CA	no	
Actinopterygii	*Eucyclogobius kristinae**	Gobiidae	no	33	32	no	
Actinopterygii	*Conodon serrifer*	Haemulidae	no	33	S of CA	yes	
Actinopterygii	*Haemulon flaviguttatum*	Haemulidae	no	33	S of CA	yes	
Actinopterygii	*Haemulopsis axillaris*	Haemulidae	no	32	S of CA	yes	
Actinopterygii	*Haemulopsis elongatus*	Haemulidae	no	32	S of CA	yes	
Actinopterygii	*Microlepidotus inornatus*	Haemulidae	no	33	S of CA	yes	
Actinopterygii	*Hexagrammos stelleri*	Hexagrammidae	no	N of CA	37	yes	
Actinopterygii	*Sectator ocyurus (Kyphosus ocyurus)*	Kyphosidae	no	35	S of CA	no	

Actinopterygii	*Labrisomus xanti*	Labrisomidae	no	34	S of CA	yes	
Actinopterygii	*Caulolatilus affinis*	Latilidae	no	34	S of CA	yes	
Actinopterygii	*Liparis nanus (Lipariscus nanus)*	Liparidae	no	N of CA	36	yes	
Actinopterygii	*Lophiodes caulinaris*	Lophiidae	no	37	S of CA	yes	
Actinopterygii	*Lophiodes spilurus*	Lophiidae	no	36	S of CA	yes	
Actinopterygii	*Lutjanus argentiventris*	Lutjanidae	no	33	S of CA	yes	
Actinopterygii	*Lutjanus colorado*	Lutjanidae	no	35	S of CA	yes	
Actinopterygii	*Lutjanus novemfasciatus*	Lutjanidae	no	35	S of CA	yes	
Actinopterygii	*Lutjanus peru*	Lutjanidae	no	32	S of CA	yes	
Actinopterygii	*Mugil curema*	Mugilidae	no	34	S of CA	yes	
Actinopterygii	*Muraena argus*	Muraenidae	no	33	S of CA	yes	
Actinopterygii	*Brotula clarkae*	Ophidiidae	no	33	S of CA	no	
Actinopterygii	*Kasatkia seigeli**	Opisthocentridae	no	39	35	yes	
Actinopterygii	*Inopsetta ischyra*	Pleuronectidae	no	N of CA	37	no	hybrid species of *Pleuronectus vetulus* and *Platichthys stellatus*
Actinopterygii	*Pomacanthus zonipectus*	Pomacanthidae	no	33	S of CA	yes	
Actinopterygii	*Abudefduf troschelii*	Pomacentridae	no	33	S of CA	yes	
Actinopterygii	*Azurina hirundo*	Pomacentridae	no	33	S of CA	yes	
Actinopterygii	*Chromis alta**	Pomacentridae	no	33	S of CA	yes	
Actinopterygii	*Stegastes leucorus*	Pomacentridae	no	33	S of CA	no	
Actinopterygii	*Heteropriacanthus carolinus (Heteropriacanthus cruentatus)*	Priacanthidae	no	33	S of CA	yes	
Actinopterygii	*Acanthocybium solandri*	Scombridae	no	34	S of CA	yes	
Actinopterygii	*Scorpaena mystes*	Scorpaenidae	no	33	S of CA	yes	
Actinopterygii	*Sebastes diaconus**	Sebastidae	no	N of CA	34	no	
Actinopterygii	*Sebastes emphaeus*	Sebastidae	no	N of CA	34	yes	
Actinopterygii	*Sebastes melanosema**	Sebastidae	no	34	S of CA	yes	
Actinopterygii	*Sebastes melanostictus*	Sebastidae	no	N of CA	32	yes	
Actinopterygii	*Sebastes moseri**	Sebastidae	no	36	S of CA	yes	
Actinopterygii	*Sebastes rufinanus**	Sebastidae	no	34	S of CA	yes	
Actinopterygii	*Cephalopholis colonus (Paranthias colonus)*	Serranidae	no	32	S of CA	yes	
Actinopterygii	*Dermatolepis dermatolepis*	Serranidae	no	33	S of CA	yes	
Actinopterygii	*Epinephelus labriformis*	Serranidae	no	32	S of CA	yes	
Actinopterygii	*Hyporthodus acanthistius*	Serranidae	no	34	S of CA	yes	
Actinopterygii	*Paralabrax auroguttatus*	Serranidae	no	34	S of CA	yes	

Actinopterygii	*Pronotogrammus multifasciatus (Anthias gordensis)*	Serranidae	no	34	S of CA	yes	
Actinopterygii	*Serranus aequidens*	Serranidae	no	33	S of CA	no	
Actinopterygii	*Esselenichthys carli**	Stichaeidae	no	36	S of CA	yes	
Actinopterygii	*Esselenichthys laurae**	Stichaeidae	no	37	S of CA	no	
Actinopterygii	*Lumpenopsis clitella**	Stichaeidae	no	33	32	no	
Actinopterygii	*Syngnathus euchrous**	Syngnathidae	no	33	S of CA	no	
Actinopterygii	*Syngnathus exilis*	Syngnathidae	no	37	S of CA	yes	
Actinopterygii	*Prionotus albirostris*	Triglidae	no	32	S of CA	no	
Actinopterygii	*Astroscopus zephyreus*	Uranoscopidae	no	33	S of CA	no	
Actinopterygii	*Eucryphycus californicus*	Zoarcidae	no	36	32	yes	
Actinopterygii	*Lycodes brevipes*	Zoarcidae	no	N of CA	34	yes	
Agnatha	*Eptatretus mcconnaugheyi**	Myxinidae	no	33	S of CA	NA	
Agnatha	*Myxine hubbsi*	Myxinidae	no	unclear	S of CA	NA	[1]: "Southern CA" to Chile
Chondrichthyes	*Alopias pelagicus*	Alopiidae	no	unclear	S of CA	yes	[1] give northern limit as "southern CA"
Chondrichthyes	*Bathyraja aleutica*	Arhynchobatidae	no	N of CA	36	yes	
Chondrichthyes	*Nasolamia velox*	Carcharhinidae	no	34	S of CA	yes	
Chondrichthyes	*Hydrolagus melanophasma**	Chimaeridae	no	36	S of CA	yes	
Chondrichthyes	*Isistius brasiliensis*	Dalatiidae	no	37	S of CA	yes	
Chondrichthyes	*Centroscyllium nigrum*	Etmopteridae	no	unclear	S of CA	yes	[1] gives northern limit as "southern California"
Chondrichthyes	*Isurus paucus*	Lamnidae	no	unclear	S of CA	yes	[1]: Only two records known from CA waters, range is unclear within California
Chondrichthyes	*Megachasma pelagios*	Megachasmidae	no	unclear	S of CA	yes	[1]: "occasional off CA". Range within California is unclear
Chondrichthyes	*Apristurus kampae**	Pentanchidae	no	N of CA	S of CA	yes	
Chondrichthyes	*Amblyraja hyperborea*	Rajidae	no	N of CA	S of CA	yes	
Chondrichthyes	*Zameus squamulosus*	Somniosidae	no	33	S of CA	yes	
Chondrichthyes	*Sphyrna lewini*	Sphyrnidae	no	34	S of CA	yes	
Actinopterygii	*Apogon pacificus*	Apogonidae	yes	33	S of CA	yes	Range extended further northward

							from 32 to 33 degree band since [3]
Actinopterygii	*Plagiotremus azaleus*	Blenniidae	yes	33	S of CA	yes	
Actinopterygii	*Engyophrys sanctilaurentii*	Bothidae	yes	32	S of CA	no	Range corrected from 33 since [3] (potentially erroneous) to 32 [1]
Actinopterygii	*Synchiropus atrilabiatus*	Callionymidae	yes	33	S of CA	yes	
Actinopterygii	*Caranx sexfasciatus*	Carangidae	yes	32	S of CA	yes	
Actinopterygii	*Caranx vinctus*	Carangidae	yes	32	S of CA	yes	
Actinopterygii	*Selene brevoortii*	Carangidae	yes	33	S of CA	yes	Range extended further northward from 32 to 33 degree band since [3]
Actinopterygii	*Ruscarius creaseri*	Cottidae	yes	36	S of CA	yes	
Actinopterygii	*Citharichthys fragilis*	Cyclopsettidae	yes	34	S of CA	no	Range extended further northward from 33 to 34 degree band since [3]
Actinopterygii	*Diodon holocanthus*	Diodontidae	yes	37	S of CA	yes	Range extended further northward from 32 to 37 degree band since [3]
Actinopterygii	*Elops affinis*	Elopidae	yes	34	S of CA	yes	
Actinopterygii	*Fistularia corneta*	Fistulariidae	yes	33	S of CA	yes	
Actinopterygii	*Rimicola cabrilloi**	Gobiesocidae	yes	34	33	no	
Actinopterygii	*Kyphosus analogus (Kyphosus vaigiensis)*	Kyphosidae	yes	36	S of CA	yes	Range extended further northward from 33 to 36 degree band since [3]
Actinopterygii	*Decodon melasma*	Labridae	yes	33	S of CA	yes	
Actinopterygii	*Nicholsina denticulata*	Labridae	yes	33	S of CA	yes	
Actinopterygii	*Lobotes pacifica*	Lobotidae	yes	33	S of CA	yes	
Actinopterygii	*Diplectrum maximum*	Serranidae	yes	33	S of CA	yes	

Actinopterygii	*Sphyraena ensis*	Sphyraenidae	yes	33	S of CA	yes	
Actinopterygii	*Anoplarchus insignis*	Stichaeidae	yes	N of CA	38	yes	
Actinopterygii	*Chirolophis decoratus*	Stichaeidae	yes	N of CA	37	yes	Range extended further southward from 40 to 37 degree band since [3]
Actinopterygii	*Sphoeroides lobatus*	Tetraodontidae	yes	33	S of CA	yes	

Table S3: Species with ambiguous information that are excluded from some analyses. Ref. [1] = Love and Passarelli 2020. Ref. [2] = Love et al. 2021. Ref. [3] = Horn et al. 2006.

**Class**	**Species (other names in databases)**	**Family**	**1970s N limit**	**1970s S limit**	**Justification**	**Used in BioGeoBEARS**	**Change in range in 2020s?**
Actinopterygii	*Ammodytes hexapterus*	Ammodytidae	N of CA	33	[2]: earlier California records referring to *A. hexapterus* are actually records for *A. personatus*; *A. hexapterus* is now a separate species restricted to N of CA.	Yes (under *A. personatus*)	No (under *A. personatus* in recent texts)
Actinopterygii	*Liparis rutteri*	Liparidae	N of CA	33	[2]: This species has been split; *L. rutteri* is no longer in CA waters. Data from 1970s refers to species now called *L. adiastolus*. *L. rutteri* is in the phylogeny, but treated as *L. adiastolus* in analyses	Yes (under outdated name *Liparis rutteri*; data for *L. adiastolus*)	No
Actinopterygii	*Melichthys niger*	Balistidae	32	S of CA	[2] claims the San Diego record dates to the 1800's, and the species has not been seen in CA waters since.	Yes (removed record from CA)	-
Actinopterygii	*Brama dussumieri*	Bramidae	-	-	[2]: only one record in California, normally a Japanese species	Excluded- cannot determine whether resident in California	Not found in 1970s dataset
Actinopterygii	*Oligoplites saurus*	Carangidae	33	S of CA	[2] contest that California records are erroneous, even though it was included in 1970's dataset	yes	Removed from CA (corrected error)
Actinopterygii	*Cetichthys parini*	Cetomimidae	-	-	[2]: only two records in California	Excluded- too little information to determine range	Not found in 1970s dataset (deep-sea fish)

Actinopterygii	*Parataeniophorus brevis*	Cetomimidae	-	-	[2] give range as "southern CA"	Excluded- cannot determine whether range extends beyond California	Not found in 1970s dataset (deep-sea fish)
Actinopterygii	*Kali falx*	Chiasmodontidae	-	-	[2]: "in northern hemisphere". Not specific enough	Excluded- too little information to determine range	Not found in 1970s dataset (deep-sea fish)
Actinopterygii	*Cottus aleuticus*	Cottidae	-	-	Sometimes found in nearshore marine waters [1] but its range in marine habitats within CA is unclear	yes	Not in 1970s dataset (mostly freshwater)
Actinopterygii	*Gasterosteus aculeatus*	Gasterosteidae	N of CA	S of CA	It appears that the 1970s dataset used its freshwater range, which is larger than its marine range (extends to S of CA). I prefer to use its marine range instead (S limit 36), and due to discrepancies it was removed from range change plot	Yes (coded using marine range)	Unclear
Actinopterygii	*Gigantactis gargantua*	Gigantactinidae	-	-	[2] give range as "southern CA"	Excluded- cannot determine whether range extends beyond California	Not found in 1970s dataset (deep-sea fish)
Actinopterygii	*Lampris guttatus*	Lampridae	N of CA	S of CA	This is no longer a valid species (see [1] and [2]) but still represented in the phylogeny. Included in BioGeoBEARS but not in range change plots	Yes (under outdated name *Lampris guttatus*)	No longer a valid species
Actinopterygii	*Linophryne racemifera*	Linophrynidae	-	-	[2] give range as "southern CA"	Excluded- cannot determine whether range extends beyond California	Not found in 1970s dataset (deep-sea fish)

Actinopterygii	*Centrobranchus nigroocellatus*	Myctophidae	-	-	[2]: only one record in California	Excluded- too little information to determine range	Not found in 1970s dataset (deep-sea fish)
Actinopterygii	*Diaphus fulgens*	Myctophidae	-	-	[2]: range poorly known	Excluded- too little information to determine range	Not found in 1970s dataset (deep-sea fish)
Actinopterygii	*Diaphus kuroshio*	Myctophidae	-	-	[2]: only one record in California	Excluded- too little information to determine range	Not found in 1970s dataset (deep-sea fish)
Actinopterygii	*Diaphus parri*	Myctophidae	-	-	[2]: only one record in California	Excluded- too little information to determine range	Not found in 1970s dataset (deep-sea fish)
Actinopterygii	*Diaphus rafinesquii*	Myctophidae	-	-	[2]: range poorly known	Excluded- too little information to determine range	Not found in 1970s dataset (deep-sea fish)
Actinopterygii	*Diaphus trachops*	Myctophidae	-	-	[2] give range as "central CA"	Excluded- cannot determine whether range extends beyond California	Not found in 1970s dataset (deep-sea fish)
Actinopterygii	*Lampanyctus nobilis*	Myctophidae	-	-	[2]: only one record in California	Excluded- too little information to determine range	Not found in 1970s dataset (deep-sea fish)
Actinopterygii	*Notoscopelus japonicus*	Myctophidae	-	-	[2]: unconfirmed west of CA.	Excluded- too little information to determine range	Not found in 1970s dataset (deep-sea fish)
Actinopterygii	*Dolopichthys longicornis*	Oneirodidae	-	-	[2] give range as "northern CA"	Excluded- cannot determine whether range extends beyond California	Not found in 1970s dataset (deep-sea fish)

Actinopterygii	*Microlophichthys microlophus*	Oneirodidae	-	-	[2] give range as “well off southern CA”	Excluded- too little information to determine range	Not found in 1970s dataset (deep-sea fish)
Actinopterygii	*Bassozetus levistomatus*	Ophidiidae	-	-	[2]: only one record in California	Excluded- too little information to determine range	Not found in 1970s dataset
Actinopterygii	*Ioichthys kashkini*	Opisthoproctidae	-	-	[2]: only one record in California	Excluded- too little information to determine range	Not found in 1970s dataset (deep-sea fish)
Actinopterygii	*Oplegnathus fasciatus*	Oplegnathidae	-	-	[2]: only entered CA as a vagrant	Excluded- not a resident in CA	Not found in 1970s dataset
Actinopterygii	*Hypomesus transpacificus*	Osmeridae	-	-	Mentioned in [1] but is predominately a freshwater species	Excluded- nonmarine	Not found in 1970s dataset (mostly freshwater)
Actinopterygii	*Allothunnus fallai*	Scombridae	33	S of CA	[2]: two isolated reports in the focal region. Seems to be vagrant in California (even though was included in 1970s dataset)	Excluded- not a resident in CA	-
Actinopterygii	*Sebastes aleutianus*	Sebastidae	N of CA	32	[2]: southern limit is northern California. [2] say this species was recently recognized, and its range limits are unclear due to confusion with other species	yes	Unclear
Actinopterygii	*Histiobranchus bathybius*	Synaphobranchidae	-	-	[2]: two records from Bering Sea and Mexico; unclear if species is found in California	Excluded- cannot determine whether found in California	Not found in 1970s dataset (deep-sea fish)
Actinopterygii	*Thaumatichthys axeli*	Thaumatichthyidae	-	-	[2]: only two records of species	Excluded- too little information to determine range	Not found in 1970s dataset (deep-sea fish)
Chondrichthyes	*Alopias pelagicus*	Alopiidae	-	-	[1] give northern limit as “southern CA”. Range within California is unclear	Yes	Not found in 1970s dataset

Chondrichthyes	*Carcharhinus limbatus*	Carcharhinidae	-	-	[2]: "unconfirmed records from California". Not mentioned in [1]	Excluded- cannot determine whether found in California	Not found in 1970s dataset
Chondrichthyes	*Carcharhinus longimanus*	Carcharhinidae	32	S of CA	[1] give northern limit as "perhaps Gaviota". If true, this would shift the range northward from 32 to 34 degrees. However, northern limit is uncertain.	yes	Unclear
Chondrichthyes	*Galeocerdo cuvier*	Carcharhinidae	33	S of CA	[1] claim this species ranges from Gulf of Alaska to Peru. However, [2] claim records in Alaska are unverified, give northern limit as "southern California".	Excluded- cannot determine whether range extends beyond California	Unclear
Chondrichthyes	*Hydrolagus trolli*	Chimaeridae	-	-	[1] claim it is normally a Central Pacific species, but it has been spotted in southern CA from ROV. These sightings are unconfirmed to be this species	Excluded- cannot determine whether found in California	Not found in 1970s dataset
Chondrichthyes	*Centroscyllium nigrum*	Etmopteridae	-	-	[1] gives northern limit as "southern California." Range within California is unclear	Yes	Not found in 1970s dataset
Chondrichthyes	*Isurus paucus*	Lamnidae	-	-	[1]: Only two records known from CA waters, range is unclear within California	Yes	Not found in 1970s dataset
Chondrichthyes	*Megachasma pelagios*	Megachasmidae	-	-	[1]: "occasional off CA". Range within California is unclear	Yes	Not found in 1970s dataset
Chondrichthyes	*Mitsukurina owstoni*	Mitsukurinidae	-	-	[1] cite one sighting in San Clemente, but otherwise not known from this region. Unclear if this is a vagrant species	Excluded- cannot determine whether found in California	Not found in 1970s dataset
Chondrichthyes	*Mobula japonica (Mobula mobular)*	Mobulidae	34	S of CA	[1] give northern limit as "central California". Cannot assign to latitudinal bands	yes	Unclear
Chondrichthyes	*Sphryna zygaena*	Sphyrnidae	37	S of CA	[1] give northern limit as "central California". Cannot assign to latitudinal bands	yes	Unclear

Chondrichthyes	*Sphyrna tiburo*	Sphyrnidae	33	S of CA	[1] claim it is historically found from San Diego to Peru. [2] claims it is formerly found in San Diego. Unclear if it is extirpated from San Diego (and thus no longer found in California waters)	Excluded- cannot determine whether still found in California	Unclear

Table S4: Region-of-origin of California ray-finned fishes as inferred from BioGeoBEARS analyses. Blue rows= southern origin species; orange rows= northern origin species; unshaded rows= species with unclear origin.

**Species**	**Family**	**Order**	**habitat**	**# maps inferring northern origin**	**# maps inferring southern origin**	**# maps indet.**	**region of origin coded in plots**	**notes**
Acanthurus xanthopterus	Acanthuridae	Acanthuriformes	demersal	0	97	3	S	
Prognathodes falcifer	Chaetodontidae	Acanthuriformes	demersal	0	97	3	S	
Lobotes pacificus	Lobotidae	Acanthuriformes	pelagic	0	91	9	S	
Luvarus imperialis	Luvaridae	Acanthuriformes	pelagic	20	73	7	S	
Pomacanthus zonipectus	Pomacanthidae	Acanthuriformes	demersal	0	96	4	S	
Acipenser medirostris	Acipenseridae	Acipenseriformes	diadromous	36	47	17	unclear	
Acipenser transmontanus	Acipenseridae	Acipenseriformes	diadromous	36	32	32	unclear	
Pseudopentaceros wheeleri	Pentacerotidae	Acropomatiformes	both demersal and pelagic	93	0	7	N	
Stereolepis gigas	Polyprionidae	Acropomatiformes	demersal	0	97	3	S	
Albula vulpes	Albulidae	Albuliformes	demersal	0	93	7	S	
Muraena argus	Muraenidae	Anguilliformes	demersal	0	99	1	S	
Myrophis vafer	Ophichthidae	Anguilliformes	demersal	0	90	10	S	
Ophichthus triserialis	Ophichthidae	Anguilliformes	demersal	11	85	4	S	
Ophichthus zophochir	Ophichthidae	Anguilliformes	demersal	3	95	2	S	
Argentina sialis	Argentinidae	Argentiniformes	demersal	58	39	3	unclear	
Atherinops affinis	Atherinopsidae	Atheriniformes	pelagic	6	70	24	S	
Atherinopsis californiensis	Atherinopsidae	Atheriniformes	pelagic	6	70	24	S	
Leuresthes tenuis	Atherinopsidae	Atheriniformes	pelagic	6	71	23	S	
Synodus lucioceps	Synodontidae	Aulopiformes	demersal	37	58	5	unclear	
Porichthys myriaster	Batrachoididae	Batrachoidiformes	demersal	0	98	2	S	
Porichthys notatus	Batrachoididae	Batrachoidiformes	demersal	0	98	2	S	
Strongylura exilis	Belonidae	Beloniformes	pelagic	0	58	42	probably S	
Cheilopogon pinnatibarbatus californicus	Exocoetidae	Beloniformes	pelagic	36	63	1	unclear	
Fodiator acutus	Exocoetidae	Beloniformes	pelagic	0	96	4	S	
Euleptorhamphus viridis	Hemiramphidae	Beloniformes	pelagic	0	88	12	S	
Hemiramphus saltator	Hemiramphidae	Beloniformes	pelagic	0	98	2	S	
Cololabis saira	Scomberesocidae	Beloniformes	pelagic	47	28	25	unclear	
Hypsoblennius gentilis	Blenniidae	Blenniiformes	demersal	0	95	5	S	

Hypsoblennius gilberti	Blenniidae	Blenniiformes	demersal	0	97	3	S	
Hypsoblennius jenkinsi	Blenniidae	Blenniiformes	demersal	0	94	6	S	
Ophioblennius steindachneri	Blenniidae	Blenniiformes	demersal	1	94	5	S	
Plagiotremus azaleus	Blenniidae	Blenniiformes	demersal	0	97	3	S	
Chaenopsis alepidota	Chaenopsidae	Blenniiformes	demersal	0	95	5	S	
Neoclinus blanchardi	Chaenopsidae	Blenniiformes	demersal	0	99	1	S	
Gibbonsia elegans	Clinidae	Blenniiformes	demersal	28	67	5	unclear	
Gibbonsia metzi	Clinidae	Blenniiformes	demersal	28	67	5	unclear	
Gibbonsia montereyensis	Clinidae	Blenniiformes	demersal	28	67	5	unclear	
Heterostichus rostratus	Clinidae	Blenniiformes	demersal	28	67	5	unclear	
Alloclinus holderi	Labrisomidae	Blenniiformes	demersal	0	94	6	S	
Labrisomus xanti	Labrisomidae	Blenniiformes	demersal	0	100	0	S	
Paraclinus integripinnis	Labrisomidae	Blenniiformes	demersal	0	97	3	S	
Synchiropus atrilabiatus	Callionymidae	Callionymiformes	demersal	0	89	11	S	
Polydactylus approximans	Polynemidae	Carangaria/misc	demersal	1	68	31	probably S	
Sphyraena argentea	Sphyraenidae	Carangaria/misc	pelagic	40	52	8	unclear	
Sphyraena ensis	Sphyraenidae	Carangaria/misc	pelagic	0	90	10	S	
Caranx caballus	Carangidae	Carangiformes	pelagic	0	97	3	S	
Caranx caninus	Carangidae	Carangiformes	pelagic	0	89	11	S	
Caranx sexfasciatus	Carangidae	Carangiformes	pelagic	0	52	48	probably S	
Caranx vinctus	Carangidae	Carangiformes	pelagic	0	89	11	S	
Chloroscombrus orqueta	Carangidae	Carangiformes	pelagic	0	91	9	S	
Decapterus muroadsi	Carangidae	Carangiformes	pelagic	0	100	0	S	
Naucrates ductor	Carangidae	Carangiformes	pelagic	40	53	7	unclear	
Selar crumenophthalmus	Carangidae	Carangiformes	pelagic	0	98	2	S	
Selene brevoortii	Carangidae	Carangiformes	demersal	0	96	4	S	
Selene peruviana	Carangidae	Carangiformes	demersal	0	95	5	S	
Seriola lalandi	Carangidae	Carangiformes	pelagic	40	54	6	unclear	
Seriola rivoliana	Carangidae	Carangiformes	pelagic	0	94	6	S	
Trachinotus paitensis	Carangidae	Carangiformes	pelagic	1	91	8	S	
Trachinotus rhodopus	Carangidae	Carangiformes	demersal	0	93	7	S	
Trachurus symmetricus	Carangidae	Carangiformes	pelagic	48	47	5	unclear	
Uraspis secunda	Carangidae	Carangiformes	demersal	0	97	3	S	
Coryphaena equiselis	Coryphaenidae	Carangiformes	pelagic	0	98	2	S	
Coryphaena hippurus	Coryphaenidae	Carangiformes	pelagic	0	98	2	S	
Echeneis naucrates	Echeneidae	Carangiformes	pelagic	0	98	2	S	
Phtheirichthys lineatus	Echeneidae	Carangiformes	pelagic	0	98	2	S	
Remora albescens	Echeneidae	Carangiformes	pelagic	0	98	2	S	

Remora australis	Echeneidae	Carangiformes	pelagic	0	98	2	S	
Remora brachyptera	Echeneidae	Carangiformes	pelagic	0	98	2	S	
Remora osteochir	Echeneidae	Carangiformes	pelagic	0	98	2	S	
Remora remora	Echeneidae	Carangiformes	pelagic	0	98	2	S	
Istiompax indica	Istiophoridae	Carangiformes	pelagic	4	91	5	S	
Istiophorus platypterus	Istiophoridae	Carangiformes	pelagic	4	91	5	S	
Kajikia audax	Istiophoridae	Carangiformes	pelagic	4	91	5	S	
Makaira mazara	Istiophoridae	Carangiformes	pelagic	4	91	5	S	
Tetrapturus angustirostris	Istiophoridae	Carangiformes	pelagic	4	91	5	S	
Xiphias gladius	Xiphiidae	Carangiformes	pelagic	4	91	5	S	
Girella nigricans	Girellidae	Centrarchiformes	demersal	46	53	1	unclear	
Kyphosus analogus	Kyphosidae	Centrarchiformes	demersal	0	93	7	S	
Kyphosus azurea	Kyphosidae	Centrarchiformes	demersal	0	94	6	S	
Medialuna californiensis	Scorpididae	Centrarchiformes	demersal	40	54	6	unclear	
Clupea harengus	Clupeidae	Clupeiformes	pelagic	51	37	12	unclear	
Harengula thrissina	Clupeidae	Clupeiformes	pelagic	0	81	19	S	
Opisthonema libertate	Clupeidae	Clupeiformes	pelagic	0	98	2	S	
Opisthonema medirastre	Clupeidae	Clupeiformes	pelagic	0	98	2	S	
Sardinops sagax	Clupeidae	Clupeiformes	pelagic	53	39	8	unclear	
Etrumeus sadina	Dussumieriidae	Clupeiformes	pelagic	0	93	7	S	
Anchoa compressa	Engraulidae	Clupeiformes	pelagic	0	88	12	S	
Anchoa delicatissima	Engraulidae	Clupeiformes	pelagic	0	64	36	probably S	
Engraulis mordax	Engraulidae	Clupeiformes	pelagic	47	46	7	unclear	
Fundulus parvipinnis	Fundulidae	Cyprinodontiformes	demersal	0	52	48	probably S	
Elops affinis	Elopidae	Elopiformes	pelagic	0	54	46	probably S	
Eucinostomus currani	Gerreidae	Eupercaria/misc	demersal	0	63	37	probably S	
Conodon serrifer	Haemulidae	Eupercaria/misc	demersal	0	99	1	S	
Haemulon flaviguttatum	Haemulidae	Eupercaria/misc	demersal	0	100	0	S	
Haemulopsis axillaris	Haemulidae	Eupercaria/misc	demersal	0	100	0	S	
Haemulopsis elongatus	Haemulidae	Eupercaria/misc	demersal	0	100	0	S	
Microlepidotus inornatus	Haemulidae	Eupercaria/misc	demersal	0	100	0	S	
Xenistius californiensis	Haemulidae	Eupercaria/misc	pelagic	1	95	4	S	
Decodon melasma	Labridae	Eupercaria/misc	demersal	1	90	9	S	
Halichoeres semicinctus	Labridae	Eupercaria/misc	demersal	0	96	4	S	
Nicholsina denticulata	Labridae	Eupercaria/misc	demersal	1	95	4	S	
Oxyjulis californica	Labridae	Eupercaria/misc	demersal	0	96	4	S	
Semicossyphus pulcher	Labridae	Eupercaria/misc	demersal	0	96	4	S	
Caulolatilus affinis	Latilidae	Eupercaria/misc	demersal	3	92	5	S	

Caulolatilus princeps	Latilidae	Eupercaria/misc	demersal	3	92	5	S	
Lutjanus argentiventris	Lutjanidae	Eupercaria/misc	demersal	0	94	6	S	
Lutjanus colorado	Lutjanidae	Eupercaria/misc	demersal	0	72	28	S	
Lutjanus novemfasciatus	Lutjanidae	Eupercaria/misc	demersal	0	72	28	S	
Lutjanus peru	Lutjanidae	Eupercaria/misc	demersal	0	97	3	S	
Heteropriacanthus cruentatus	Priacanthidae	Eupercaria/misc	demersal	0	100	0	S	
Pristigenys serrula	Priacanthidae	Eupercaria/misc	demersal	40	52	8	unclear	
Atractoscion nobilis	Sciaenidae	Eupercaria/misc	demersal	44	52	4	unclear	
Cheilotrema saturnum	Sciaenidae	Eupercaria/misc	demersal	0	96	4	S	
Cynoscion parvipinnis	Sciaenidae	Eupercaria/misc	demersal	0	91	9	S	
Genyonemus lineatus	Sciaenidae	Eupercaria/misc	demersal	9	82	9	S	
Menticirrhus undulatus	Sciaenidae	Eupercaria/misc	demersal	0	88	12	S	
Roncador stearnsii	Sciaenidae	Eupercaria/misc	demersal	9	82	9	S	
Seriphus politus	Sciaenidae	Eupercaria/misc	demersal	9	82	9	S	
Umbrina roncador	Sciaenidae	Eupercaria/misc	demersal	0	95	5	S	
Calamus brachysomus	Sparidae	Eupercaria/misc	demersal	0	96	4	S	
Gadus macrocephalus	Gadidae	Gadiformes	both demersal and pelagic	99	0	1	N	
Microgadus proximus	Gadidae	Gadiformes	demersal	98	0	2	N	
Theragra chalcogramma	Gadidae	Gadiformes	both demersal and pelagic	99	0	1	N	
Coryphaenoides acrolepis	Macrouridae	Gadiformes	both demersal and pelagic	97	0	3	N	
Merluccius productus	Merlucciidae	Gadiformes	pelagic	43	55	2	unclear	
Antimora microlepis	Moridae	Gadiformes	demersal	72	20	8	N	
Physiculus rastrelliger	Moridae	Gadiformes	demersal	46	48	6	unclear	
Gobiesox maeandricus	Gobiesocidae	Gobiesociformes	demersal	0	100	0	S	
Gobiesox rhessodon	Gobiesocidae	Gobiesociformes	demersal	0	100	0	S	
Dormitator latifrons	Eleotridae	Gobiiformes	demersal	0	73	27	S	
Clevelandia ios	Gobiidae	Gobiiformes	demersal	0	89	11	S	
Ctenogobius sagittula	Gobiidae	Gobiiformes	demersal	0	79	21	S	
Eucyclogobius newberryi	Gobiidae	Gobiiformes	demersal	0	92	8	S	
Gillichthys mirabilis	Gobiidae	Gobiiformes	demersal	0	93	7	S	
Lepidogobius lepidus	Gobiidae	Gobiiformes	demersal	0	92	8	S	
Lethops connectens	Gobiidae	Gobiiformes	demersal	0	89	11	S	
Lythrypnus dalli	Gobiidae	Gobiiformes	demersal	0	94	6	S	
Lythrypnus zebra	Gobiidae	Gobiiformes	demersal	0	97	3	S	
Quietula y-cauda	Gobiidae	Gobiiformes	demersal	0	89	11	S	

Rhinogobiops nicholsii	Gobiidae	Gobiiformes	demersal	48	44	8	unclear	
Typhlogobius californiensis	Gobiidae	Gobiiformes	demersal	0	92	8	S	
Chanos chanos	Chanidae	Gonorynchiformes	pelagic	3	79	18	S	
Apogon guadalupensis	Apogonidae	Kurtiformes	demersal	0	100	0	S	
Apogon pacificus	Apogonidae	Kurtiformes	demersal	0	100	0	S	
Lophotus capellei	Lophotidae	Lampriformes	pelagic	18	15	67	unclear	
Trachipterus altivelis	Trachipteridae	Lampriformes	pelagic	47	47	6	unclear	
Zu cristatus	Trachipteridae	Lampriformes	pelagic	1	93	6	S	
Fowlerichthys avalonis	Antennariidae	Lophiiformes	demersal	0	94	6	S	
Lophiodes caulinaris	Lophiidae	Lophiiformes	demersal	0	96	4	S	
Lophiodes spilurus	Lophiidae	Lophiiformes	demersal	0	96	4	S	
Zalieutes elater	Ogcocephalidae	Lophiiformes	demersal	49	41	10	unclear	
Mugil cephalus	Mugilidae	Mugiliformes	diadromous	0	67	33	probably S	
Mugil curema	Mugilidae	Mugiliformes	pelagic	0	61	39	probably S	
Pseudupeneus grandisquamis	Mullidae	Mulliformes	demersal	0	98	2	S	
Brosmophycis marginata	Bythitidae	Ophidiiformes	demersal	49	46	5	unclear	
Chilara taylori	Ophidiidae	Ophidiiformes	demersal	53	41	6	unclear	
Allosmerus elongatus	Osmeridae	Osmeriformes	demersal	87	0	13	N	
Hypomesus pretiosus	Osmeridae	Osmeriformes	demersal	68	0	32	probably N	
Spirinchus starksi	Osmeridae	Osmeriformes	demersal	87	0	13	N	
Spirinchus thaleichthys	Osmeridae	Osmeriformes	diadromous	87	0	13	N	
Thaleichthys pacificus	Osmeridae	Osmeriformes	diadromous	87	0	13	N	
Amphistichus koelzi	Embiotocidae	Ovalentaria/misc	demersal	8	82	10	S	
Amphistichus rhodoterus	Embiotocidae	Ovalentaria/misc	demersal	8	82	10	S	
Brachyistius frenatus	Embiotocidae	Ovalentaria/misc	demersal	8	82	10	S	
Cymatogaster aggregata	Embiotocidae	Ovalentaria/misc	demersal	8	82	10	S	
Embiotoca jacksoni	Embiotocidae	Ovalentaria/misc	demersal	8	82	10	S	
Embiotoca lateralis	Embiotocidae	Ovalentaria/misc	demersal	8	82	10	S	
Hyperprosopon anale	Embiotocidae	Ovalentaria/misc	pelagic	8	82	10	S	
Hyperprosopon argenteum	Embiotocidae	Ovalentaria/misc	demersal	8	82	10	S	
Hyperprosopon ellipticum	Embiotocidae	Ovalentaria/misc	demersal	8	82	10	S	
Hypsurus caryi	Embiotocidae	Ovalentaria/misc	demersal	8	82	10	S	
Micrometrus minimus	Embiotocidae	Ovalentaria/misc	demersal	8	82	10	S	
Phanerodon atripes	Embiotocidae	Ovalentaria/misc	demersal	8	82	10	S	
Phanerodon furcatus	Embiotocidae	Ovalentaria/misc	demersal	8	82	10	S	
Rhacochilus toxotes	Embiotocidae	Ovalentaria/misc	demersal	8	82	10	S	
Rhacochilus vacca	Embiotocidae	Ovalentaria/misc	demersal	8	82	10	S	
Zalembius rosaceus	Embiotocidae	Ovalentaria/misc	demersal	8	82	10	S	

Abudefduf troschelii	Pomacentridae	Ovalentaria/misc	demersal	0	93	7	S	
Azurina hirundo	Pomacentridae	Ovalentaria/misc	demersal	0	100	0	S	
Chromis alta	Pomacentridae	Ovalentaria/misc	demersal	0	95	5	S	
Chromis punctipinnis	Pomacentridae	Ovalentaria/misc	demersal	0	96	4	S	
Hypsypops rubicundus	Pomacentridae	Ovalentaria/misc	demersal	0	99	1	S	
Agonopsis sterletus	Agonidae	Perciformes/Cottoidei	demersal	98	0	2	N	
Agonopsis vulsa	Agonidae	Perciformes/Cottoidei	demersal	98	0	2	N	
Anoplagonus inermis	Agonidae	Perciformes/Cottoidei	demersal	100	0	0	N	
Bathyagonus alascanus	Agonidae	Perciformes/Cottoidei	demersal	98	0	2	N	
Bathyagonus infraspinatus	Agonidae	Perciformes/Cottoidei	demersal	98	0	2	N	
Bathyagonus nigripinnis	Agonidae	Perciformes/Cottoidei	demersal	98	0	2	N	
Bathyagonus pentacanthus	Agonidae	Perciformes/Cottoidei	demersal	98	0	2	N	
Blepsias cirrhosus	Agonidae	Perciformes/Cottoidei	demersal	100	0	0	N	
Nautichthys oculofasciatus	Agonidae	Perciformes/Cottoidei	demersal	96	1	3	N	
Odontopyxis trispinosa	Agonidae	Perciformes/Cottoidei	demersal	98	0	2	N	
Podothecus accipenserinus	Agonidae	Perciformes/Cottoidei	demersal	100	0	0	N	
Stellerina xyosterna	Agonidae	Perciformes/Cottoidei	demersal	98	0	2	N	
Xeneretmus latifrons	Agonidae	Perciformes/Cottoidei	demersal	98	0	2	N	
Xeneretmus leiops	Agonidae	Perciformes/Cottoidei	demersal	98	0	2	N	
Anoplopoma fimbria	Anoplopomatidae	Perciformes/Cottoidei	demersal	95	2	3	N	
Erilepis zonifer	Anoplopomatidae	Perciformes/Cottoidei	demersal	95	2	3	N	
Artedius corallinus	Cottidae	Perciformes/Cottoidei	demersal	100	0	0	N	
Artedius fenestralis	Cottidae	Perciformes/Cottoidei	demersal	100	0	0	N	
Artedius harringtoni	Cottidae	Perciformes/Cottoidei	demersal	100	0	0	N	
Artedius lateralis	Cottidae	Perciformes/Cottoidei	demersal	100	0	0	N	
Artedius notospilotus	Cottidae	Perciformes/Cottoidei	demersal	100	0	0	N	
Chitonotus pugetensis	Cottidae	Perciformes/Cottoidei	demersal	100	0	0	N	
Clinocottus acuticeps	Cottidae	Perciformes/Cottoidei	demersal	100	0	0	N	
Clinocottus analis	Cottidae	Perciformes/Cottoidei	demersal	100	0	0	N	
Clinocottus embryum	Cottidae	Perciformes/Cottoidei	demersal	100	0	0	N	
Clinocottus globiceps	Cottidae	Perciformes/Cottoidei	demersal	100	0	0	N	
Clinocottus recalvus	Cottidae	Perciformes/Cottoidei	demersal	100	0	0	N	
Cottus asper	Cottidae	Perciformes/Cottoidei	diadromous	57	0	43	probably N	
Enophrys bison	Cottidae	Perciformes/Cottoidei	demersal	79	0	21	N	
Enophrys taurina	Cottidae	Perciformes/Cottoidei	demersal	79	0	21	N	
Hemilepidotus hemilepidotus	Cottidae	Perciformes/Cottoidei	demersal	99	0	1	N	
Icelinus burchami	Cottidae	Perciformes/Cottoidei	demersal	100	0	0	N	
Icelinus cavifrons	Cottidae	Perciformes/Cottoidei	demersal	99	1	0	N	

Icelinus filamentosus	Cottidae	Perciformes/Cottoidei	demersal	99	1	0	N	
Icelinus fimbriatus	Cottidae	Perciformes/Cottoidei	demersal	100	0	0	N	
Icelinus oculatus	Cottidae	Perciformes/Cottoidei	demersal	100	0	0	N	
Icelinus quadriseriatus	Cottidae	Perciformes/Cottoidei	demersal	99	1	0	N	
Icelinus tenuis	Cottidae	Perciformes/Cottoidei	demersal	99	1	0	N	
Jordania zonope	Cottidae	Perciformes/Cottoidei	demersal	97	1	2	N	
Leiocottus hirundo	Cottidae	Perciformes/Cottoidei	demersal	100	0	0	N	
Leptocottus armatus	Cottidae	Perciformes/Cottoidei	demersal	69	10	21	probably N	
Oligocottus maculosus	Cottidae	Perciformes/Cottoidei	demersal	100	0	0	N	
Oligocottus rimensis	Cottidae	Perciformes/Cottoidei	demersal	100	0	0	N	
Oligocottus rubellio	Cottidae	Perciformes/Cottoidei	demersal	100	0	0	N	
Oligocottus snyderi	Cottidae	Perciformes/Cottoidei	demersal	100	0	0	N	
Orthonopias triacis	Cottidae	Perciformes/Cottoidei	demersal	100	0	0	N	
Radulinus asprellus	Cottidae	Perciformes/Cottoidei	demersal	100	0	0	N	
Radulinus boleoides	Cottidae	Perciformes/Cottoidei	demersal	100	0	0	N	
Ruscarius creaseri	Cottidae	Perciformes/Cottoidei	demersal	100	0	0	N	
Scorpaenichthys marmoratus	Cottidae	Perciformes/Cottoidei	demersal	97	1	2	N	
Zesticelus profundorum	Cottidae	Perciformes/Cottoidei	demersal	98	2	0	N	
Hexagrammos decagrammus	Hexagrammidae	Perciformes/Cottoidei	demersal	95	2	3	N	
Hexagrammos lagocephalus	Hexagrammidae	Perciformes/Cottoidei	demersal	95	2	3	N	
Hexagrammos stelleri	Hexagrammidae	Perciformes/Cottoidei	demersal	95	2	3	N	
Ophiodon elongatus	Hexagrammidae	Perciformes/Cottoidei	demersal	95	2	3	N	
Pleurogrammus monopterygius	Hexagrammidae	Perciformes/Cottoidei	demersal	95	2	3	N	
Careproctus melanurus	Liparidae	Perciformes/Cottoidei	demersal	96	1	3	N	
Liparis florae	Liparidae	Perciformes/Cottoidei	demersal	96	1	3	N	
Liparis fucensis	Liparidae	Perciformes/Cottoidei	demersal	98	0	2	N	
Liparis mucosus	Liparidae	Perciformes/Cottoidei	demersal	96	1	3	N	
Liparis pulchellus	Liparidae	Perciformes/Cottoidei	demersal	96	1	3	N	
Liparis rutteri	Liparidae	Perciformes/Cottoidei	demersal	96	1	3	N	
Lipariscus nanus	Liparidae	Perciformes/Cottoidei	demersal	96	1	3	N	
Rhamphocottus richardsonii	Rhamphocottidae	Perciformes/Cottoidei	demersal	97	1	2	N	
Oxylebius pictus	Zaniolepididae	Perciformes/Cottoidei	demersal	95	2	3	N	
Zaniolepis frenata	Zaniolepididae	Perciformes/Cottoidei	demersal	95	2	3	N	
Zaniolepis latipinnis	Zaniolepididae	Perciformes/Cottoidei	demersal	95	2	3	N	
Aulorhynchus flavidus	Aulorhynchidae	Perciformes/Gasterosteoidei	pelagic	85	7	8	N	
Scorpaena guttata	Scorpaenidae	Perciformes/Scorpaenoidei	demersal	0	93	7	S	
Scorpaena mystes	Scorpaenidae	Perciformes/Scorpaenoidei	demersal	0	96	4	S	

Scorpaenodes xyris	Scorpaenidae	Perciformes/Scorpaenoidei	demersal	0	93	7	S	
Sebastes alutus	Sebastidae	Perciformes/Scorpaenoidei	demersal	96	0	4	N	
Sebastes atrovirens	Sebastidae	Perciformes/Scorpaenoidei	demersal	96	0	4	N	
Sebastes auriculatus	Sebastidae	Perciformes/Scorpaenoidei	demersal	96	0	4	N	
Sebastes aurora	Sebastidae	Perciformes/Scorpaenoidei	demersal	96	0	4	N	
Sebastes babcocki	Sebastidae	Perciformes/Scorpaenoidei	demersal	96	0	4	N	
Sebastes borealis	Sebastidae	Perciformes/Scorpaenoidei	demersal	96	0	4	N	
Sebastes brevispinis	Sebastidae	Perciformes/Scorpaenoidei	demersal	96	0	4	N	
Sebastes carnatus	Sebastidae	Perciformes/Scorpaenoidei	demersal	96	0	4	N	
Sebastes caurinus	Sebastidae	Perciformes/Scorpaenoidei	demersal	96	0	4	N	
Sebastes chlorostictus	Sebastidae	Perciformes/Scorpaenoidei	demersal	96	0	4	N	
Sebastes chrysomelas	Sebastidae	Perciformes/Scorpaenoidei	demersal	96	0	4	N	
Sebastes constellatus	Sebastidae	Perciformes/Scorpaenoidei	demersal	96	0	4	N	
Sebastes crameri	Sebastidae	Perciformes/Scorpaenoidei	demersal	100	0	0	N	
Sebastes dallii	Sebastidae	Perciformes/Scorpaenoidei	demersal	96	0	4	N	
Sebastes diploproa	Sebastidae	Perciformes/Scorpaenoidei	demersal	96	0	4	N	
Sebastes elongatus	Sebastidae	Perciformes/Scorpaenoidei	demersal	96	0	4	N	
Sebastes emphaeus	Sebastidae	Perciformes/Scorpaenoidei	demersal	96	0	4	N	
Sebastes ensifer	Sebastidae	Perciformes/Scorpaenoidei	demersal	96	0	4	N	
Sebastes entomelas	Sebastidae	Perciformes/Scorpaenoidei	demersal	96	0	4	N	
Sebastes eos	Sebastidae	Perciformes/Scorpaenoidei	demersal	96	0	4	N	
Sebastes flavidus	Sebastidae	Perciformes/Scorpaenoidei	pelagic	96	0	4	N	
Sebastes gilli	Sebastidae	Perciformes/Scorpaenoidei	demersal	96	0	4	N	
Sebastes helvomaculatus	Sebastidae	Perciformes/Scorpaenoidei	demersal	96	0	4	N	
Sebastes hopkinsi	Sebastidae	Perciformes/Scorpaenoidei	demersal	96	0	4	N	
Sebastes jordani	Sebastidae	Perciformes/Scorpaenoidei	demersal	96	0	4	N	
Sebastes lentiginosus	Sebastidae	Perciformes/Scorpaenoidei	demersal	96	0	4	N	
Sebastes macdonaldi	Sebastidae	Perciformes/Scorpaenoidei	demersal	96	0	4	N	
Sebastes maliger	Sebastidae	Perciformes/Scorpaenoidei	demersal	96	0	4	N	
Sebastes melanops	Sebastidae	Perciformes/Scorpaenoidei	demersal	96	0	4	N	
Sebastes melanosema	Sebastidae	Perciformes/Scorpaenoidei	demersal	95	1	4	N	
Sebastes melanostictus	Sebastidae	Perciformes/Scorpaenoidei	demersal	96	0	4	N	
Sebastes melanostomus	Sebastidae	Perciformes/Scorpaenoidei	demersal	96	0	4	N	
Sebastes miniatus	Sebastidae	Perciformes/Scorpaenoidei	demersal	96	0	4	N	
Sebastes moseri	Sebastidae	Perciformes/Scorpaenoidei	demersal	96	0	4	N	
Sebastes mystinus	Sebastidae	Perciformes/Scorpaenoidei	pelagic	96	0	4	N	
Sebastes nebulosus	Sebastidae	Perciformes/Scorpaenoidei	demersal	96	0	4	N	
Sebastes nigrocinctus	Sebastidae	Perciformes/Scorpaenoidei	demersal	96	0	4	N	

Sebastes ovalis	Sebastidae	Perciformes/Scorpaenoidei	demersal	96	0	4	N	
Sebastes paucispinis	Sebastidae	Perciformes/Scorpaenoidei	demersal	96	0	4	N	
Sebastes phillipsi	Sebastidae	Perciformes/Scorpaenoidei	demersal	96	0	4	N	
Sebastes pinniger	Sebastidae	Perciformes/Scorpaenoidei	demersal	96	0	4	N	
Sebastes proriger	Sebastidae	Perciformes/Scorpaenoidei	demersal	96	0	4	N	
Sebastes rastrelliger	Sebastidae	Perciformes/Scorpaenoidei	demersal	96	0	4	N	
Sebastes reedi	Sebastidae	Perciformes/Scorpaenoidei	demersal	100	0	0	N	
Sebastes rosenblatti	Sebastidae	Perciformes/Scorpaenoidei	demersal	96	0	4	N	
Sebastes ruberrimus	Sebastidae	Perciformes/Scorpaenoidei	demersal	96	0	4	N	
Sebastes rubrivinctus	Sebastidae	Perciformes/Scorpaenoidei	demersal	96	0	4	N	
Sebastes rufinanus	Sebastidae	Perciformes/Scorpaenoidei	demersal	96	0	4	N	
Sebastes rufus	Sebastidae	Perciformes/Scorpaenoidei	demersal	96	0	4	N	
Sebastes saxicola	Sebastidae	Perciformes/Scorpaenoidei	demersal	96	0	4	N	
Sebastes semicinctus	Sebastidae	Perciformes/Scorpaenoidei	demersal	96	0	4	N	
Sebastes serranoides	Sebastidae	Perciformes/Scorpaenoidei	demersal	96	0	4	N	
Sebastes serriceps	Sebastidae	Perciformes/Scorpaenoidei	demersal	96	0	4	N	
Sebastes simulator	Sebastidae	Perciformes/Scorpaenoidei	demersal	96	0	4	N	
Sebastes umbrosus	Sebastidae	Perciformes/Scorpaenoidei	demersal	96	0	4	N	
Sebastes wilsoni	Sebastidae	Perciformes/Scorpaenoidei	demersal	96	0	4	N	
Sebastes zacentrus	Sebastidae	Perciformes/Scorpaenoidei	demersal	96	0	4	N	
Sebastolobus alascanus	Sebastidae	Perciformes/Scorpaenoidei	demersal	100	0	0	N	
Sebastolobus altivelis	Sebastidae	Perciformes/Scorpaenoidei	demersal	100	0	0	N	
Bellator xenisma	Triglidae	Perciformes/Scorpaenoidei	demersal	0	97	3	S	
Prionotus stephanophrys	Triglidae	Perciformes/Scorpaenoidei	demersal	37	56	7	unclear	
Dermatolepis dermatolepis	Serranidae	Perciformes/Serranoidei	demersal	0	97	3	S	
Diplectrum maximum	Serranidae	Perciformes/Serranoidei	demersal	0	99	1	S	
Epinephelus analogus	Serranidae	Perciformes/Serranoidei	demersal	0	94	6	S	
Epinephelus labriformis	Serranidae	Perciformes/Serranoidei	demersal	0	96	4	S	
Hemanthias signifer	Serranidae	Perciformes/Serranoidei	demersal	0	90	10	S	
Hyporthodus acanthistius	Serranidae	Perciformes/Serranoidei	demersal	0	98	2	S	
Hyporthodus niphobles	Serranidae	Perciformes/Serranoidei	demersal	0	98	2	S	
Mycteroperca jordani	Serranidae	Perciformes/Serranoidei	demersal	0	100	0	S	
Mycteroperca xenarcha	Serranidae	Perciformes/Serranoidei	demersal	0	97	3	S	
Paralabrax auroguttatus	Serranidae	Perciformes/Serranoidei	demersal	0	96	4	S	
Paralabrax clathratus	Serranidae	Perciformes/Serranoidei	demersal	0	96	4	S	
Paralabrax maculatofasciatus	Serranidae	Perciformes/Serranoidei	demersal	0	96	4	S	
Paralabrax nebulifer	Serranidae	Perciformes/Serranoidei	demersal	0	96	4	S	
Paranthias colonus	Serranidae	Perciformes/Serranoidei	pelagic	1	94	5	S	

Pronotogrammus multifasciatus	Serranidae	Perciformes/Serranoidei	demersal	0	97	3	S	
Ammodytes personatus	Ammodytidae	Perciformes/Uranoscopoidei	demersal	96	0	4	N	
Trichodon trichodon	Trichodontidae	Perciformes/Uranoscopoidei	demersal	96	1	3	N	
Kathetostoma averruncus	Uranoscopidae	Perciformes/Uranoscopoidei	demersal	0	95	5	S	
Anarrhichthys ocellatus	Anarhichadidae	Perciformes/Zoarcoidei	demersal	97	3	0	N	
Rathbunella hypoplecta	Bathymasteridae	Perciformes/Zoarcoidei	demersal	95	2	3	N	
Ronquilus jordani	Bathymasteridae	Perciformes/Zoarcoidei	demersal	95	2	3	N	
Cebidichthys violaceus	Cebidichthyidae	Perciformes/Zoarcoidei	demersal	95	2	3	N	
Cryptacanthodes aleutensis	Cryptacanthodidae	Perciformes/Zoarcoidei	demersal	95	2	3	N	
Cryptacanthodes giganteus	Cryptacanthodidae	Perciformes/Zoarcoidei	demersal	95	2	3	N	
Lumpenus sagitta	Lumpenidae	Perciformes/Zoarcoidei	demersal	100	0	0	N	
Poroclinus rothrocki	Lumpenidae	Perciformes/Zoarcoidei	demersal	95	2	3	N	
Kasatkia seigeli	Opisthocentridae	Perciformes/Zoarcoidei	demersal	33	2	65	probably N	
Apodichthys flavidus	Pholidae	Perciformes/Zoarcoidei	demersal	95	2	3	N	
Apodichthys fucorum	Pholidae	Perciformes/Zoarcoidei	demersal	95	2	3	N	
Pholis clemensi	Pholidae	Perciformes/Zoarcoidei	demersal	95	2	3	N	
Pholis laeta	Pholidae	Perciformes/Zoarcoidei	demersal	95	2	3	N	
Pholis ornata	Pholidae	Perciformes/Zoarcoidei	demersal	95	2	3	N	
Anoplarchus insignis	Stichaeidae	Perciformes/Zoarcoidei	demersal	95	2	3	N	
Anoplarchus purpurescens	Stichaeidae	Perciformes/Zoarcoidei	demersal	95	2	3	N	
Chirolophis decoratus	Stichaeidae	Perciformes/Zoarcoidei	demersal	97	0	3	N	
Chirolophis nugator	Stichaeidae	Perciformes/Zoarcoidei	demersal	97	0	3	N	
Esselenichthys carli	Stichaeidae	Perciformes/Zoarcoidei	demersal	95	2	3	N	
Xiphister mucosus	Stichaeidae	Perciformes/Zoarcoidei	demersal	95	2	3	N	
Zaprora silenus	Zaproridae	Perciformes/Zoarcoidei	demersal	95	2	3	N	
Bothrocara brunneum	Zoarcidae	Perciformes/Zoarcoidei	demersal	73	0	27	N	
Bothrocara molle	Zoarcidae	Perciformes/Zoarcoidei	demersal	73	0	27	N	
Eucryphycus californicus	Zoarcidae	Perciformes/Zoarcoidei	demersal	73	0	27	N	
Lycenchelys crotalinus	Zoarcidae	Perciformes/Zoarcoidei	demersal	73	0	27	N	
Lycodapus fierasfer	Zoarcidae	Perciformes/Zoarcoidei	both demersal and pelagic	73	0	27	N	
Lycodapus mandibularis	Zoarcidae	Perciformes/Zoarcoidei	pelagic	74	0	26	N	
Lycodes brevipes	Zoarcidae	Perciformes/Zoarcoidei	demersal	97	0	3	N	
Lycodes cortezianus	Zoarcidae	Perciformes/Zoarcoidei	demersal	73	0	27	N	
Lycodes diapterus	Zoarcidae	Perciformes/Zoarcoidei	demersal	75	0	25	N	

Lycodes pacificus	Zoarcidae	Perciformes/Zoarcoidei	demersal	73	0	27	N	
Melanostigma pammelas	Zoarcidae	Perciformes/Zoarcoidei	pelagic	45	51	4	unclear	
Citharichthys sordidus	Cyclopsettidae	Pleuronectiformes	demersal	0	100	0	S	
Citharichthys stigmaeus	Cyclopsettidae	Pleuronectiformes	demersal	1	99	0	S	
Citharichthys xanthostigma	Cyclopsettidae	Pleuronectiformes	demersal	0	100	0	S	
Symphurus atricaudus	Cynoglossidae	Pleuronectiformes	demersal	46	49	5	unclear	
Hippoglossina stomata	Paralichthyidae	Pleuronectiformes	demersal	1	96	3	S	
Paralichthys californicus	Paralichthyidae	Pleuronectiformes	demersal	94	0	6	N	
Xystreurys liolepis	Paralichthyidae	Pleuronectiformes	demersal	0	96	4	S	
Atheresthes stomias	Pleuronectidae	Pleuronectiformes	demersal	55	41	4	unclear	
Eopsetta jordani	Pleuronectidae	Pleuronectiformes	demersal	50	46	4	unclear	
Glyptocephalus zachirus	Pleuronectidae	Pleuronectiformes	demersal	50	46	4	unclear	
Hippoglossoides elassodon	Pleuronectidae	Pleuronectiformes	demersal	97	0	3	N	
Hippoglossus stenolepis	Pleuronectidae	Pleuronectiformes	demersal	50	46	4	unclear	
Hypsopsetta guttulata	Pleuronectidae	Pleuronectiformes	demersal	50	46	4	unclear	
Isopsetta isolepis	Pleuronectidae	Pleuronectiformes	demersal	93	7	0	N	
Lepidopsetta bilineata	Pleuronectidae	Pleuronectiformes	demersal	93	7	0	N	
Lyopsetta exilis	Pleuronectidae	Pleuronectiformes	demersal	50	46	4	unclear	
Microstomus pacificus	Pleuronectidae	Pleuronectiformes	demersal	50	46	4	unclear	
Parophrys vetulus	Pleuronectidae	Pleuronectiformes	demersal	93	7	0	N	
Platichthys stellatus	Pleuronectidae	Pleuronectiformes	demersal	65	0	35	probably N	
Pleuronichthys coenosus	Pleuronectidae	Pleuronectiformes	demersal	50	46	4	unclear	
Pleuronichthys decurrens	Pleuronectidae	Pleuronectiformes	demersal	50	46	4	unclear	
Pleuronichthys ritteri	Pleuronectidae	Pleuronectiformes	demersal	50	46	4	unclear	
Pleuronichthys verticalis	Pleuronectidae	Pleuronectiformes	demersal	50	46	4	unclear	
Psettichthys melanostictus	Pleuronectidae	Pleuronectiformes	demersal	93	7	0	N	
Reinhardtius hippoglossoides	Pleuronectidae	Pleuronectiformes	demersal	50	46	4	unclear	
Oncorhynchus clarkii	Salmonidae	Salmoniformes	diadromous	59	0	41	probably N	
Oncorhynchus gorbuscha	Salmonidae	Salmoniformes	diadromous	59	0	41	probably N	
Oncorhynchus keta	Salmonidae	Salmoniformes	diadromous	59	0	41	probably N	
Oncorhynchus kisutch	Salmonidae	Salmoniformes	diadromous	59	0	41	probably N	
Oncorhynchus mykiss	Salmonidae	Salmoniformes	diadromous	61	1	38	probably N	
Oncorhynchus nerka	Salmonidae	Salmoniformes	diadromous	59	0	41	probably N	
Oncorhynchus tshawytscha	Salmonidae	Salmoniformes	diadromous	59	0	41	probably N	
Brama japonica	Bramidae	Scombriformes	pelagic	42	52	6	unclear	
Taractichthys steindachneri	Bramidae	Scombriformes	pelagic	9	87	4	S	
Icichthys lockingtoni	Centrolophidae	Scombriformes	pelagic	47	51	2	unclear	
Gempylus serpens	Gempylidae	Scombriformes	pelagic	1	96	3	S	

Lepidocybium flavobrunneum	Gempylidae	Scombriformes	pelagic	0	92	8	S	
Ruvettus pretiosus	Gempylidae	Scombriformes	demersal	1	94	5	S	
Icosteus aenigmaticus	Icosteidae	Scombriformes	demersal	91	1	8	N	
Acanthocybium solandri	Scombridae	Scombriformes	pelagic	1	93	6	S	
Auxis rochei rochei	Scombridae	Scombriformes	pelagic	1	93	6	S	
Auxis thazard thazard	Scombridae	Scombriformes	pelagic	1	93	6	S	
Euthynnus affinis	Scombridae	Scombriformes	pelagic	1	93	6	S	
Euthynnus lineatus	Scombridae	Scombriformes	pelagic	1	93	6	S	
Katsuwonus pelamis	Scombridae	Scombriformes	pelagic	1	93	6	S	
Sarda chiliensis chiliensis	Scombridae	Scombriformes	pelagic	2	93	5	S	
Scomber japonicus	Scombridae	Scombriformes	pelagic	40	53	7	unclear	
Scomberomorus concolor	Scombridae	Scombriformes	pelagic	0	92	8	S	
Scomberomorus sierra	Scombridae	Scombriformes	pelagic	0	92	8	S	
Thunnus alalunga	Scombridae	Scombriformes	pelagic	1	93	6	S	
Thunnus albacares	Scombridae	Scombriformes	pelagic	1	93	6	S	
Thunnus obesus	Scombridae	Scombriformes	pelagic	1	93	6	S	
Thunnus orientalis	Scombridae	Scombriformes	pelagic	1	93	6	S	
Peprilus simillimus	Stromateidae	Scombriformes	demersal	43	45	12	unclear	
Tetragonurus cuvieri	Tetragonuridae	Scombriformes	pelagic	5	94	1	S	
Assurger anzac	Trichiuridae	Scombriformes	demersal	0	96	4	S	
Trichiurus lepturus	Trichiuridae	Scombriformes	demersal	0	95	5	S	
Bagre panamensis	Ariidae	Siluriformes	demersal	0	80	20	S	
Macroramphosus gracilis	Centriscidae	Syngnathiformes	both demersal and pelagic	1	94	5	S	
Fistularia commersonii	Fistulariidae	Syngnathiformes	demersal	1	92	7	S	
Fistularia corneta	Fistulariidae	Syngnathiformes	demersal	1	92	7	S	
Syngnathus auliscus	Syngnathidae	Syngnathiformes	demersal	0	87	13	S	
Syngnathus californiensis	Syngnathidae	Syngnathiformes	demersal	0	87	13	S	
Syngnathus exilis	Syngnathidae	Syngnathiformes	demersal	0	87	13	S	
Syngnathus leptorhynchus	Syngnathidae	Syngnathiformes	demersal	0	87	13	S	
Balistes polylepis	Balistidae	Tetraodontiformes	demersal	49	47	4	unclear	
Xanthichthys mento	Balistidae	Tetraodontiformes	demersal	0	96	4	S	
Chilomycterus reticulatus	Diodontidae	Tetraodontiformes	demersal	0	97	3	S	
Diodon holocanthus	Diodontidae	Tetraodontiformes	demersal	0	97	3	S	
Diodon hystrix	Diodontidae	Tetraodontiformes	demersal	0	97	3	S	
Mola mola	Molidae	Tetraodontiformes	pelagic	2	94	4	S	
Ranzania laevis	Molidae	Tetraodontiformes	pelagic	2	94	4	S	
Lactoria diaphana	Ostraciidae	Tetraodontiformes	demersal	0	93	7	S	

Lagocephalus lagocephalus lagocephalus	Tetraodontidae	Tetraodontiformes	pelagic	0	97	3	S	
Sphoeroides annulatus	Tetraodontidae	Tetraodontiformes	demersal	0	91	9	S	
Sphoeroides lobatus	Tetraodontidae	Tetraodontiformes	demersal	0	81	19	S	
Allocyttus folletti	Oreosomatidae	Zeiformes	demersal	93	0	7	N	
Zenopsis nebulosa	Zeidae	Zeiformes	pelagic	2	1	97	probably N	
Howella brodiei	Howellidae	Acropomatiformes	pelagic	49	49	2	unclear	Fig. S1 only
Alepocephalus tenebrosus	Alepocephalidae	Alepocephaliformes	demersal	40	45	15	unclear	Fig. S1 only
Bajacalifornia burragei	Alepocephalidae	Alepocephaliformes	demersal	40	45	15	unclear	Fig. S1 only
Bathylaco nigricans	Alepocephalidae	Alepocephaliformes	pelagic	40	45	15	unclear	Fig. S1 only
Talismania bifurcata	Alepocephalidae	Alepocephaliformes	demersal	40	45	15	unclear	Fig. S1 only
Holtbyrnia latifrons	Platytroctidae	Alepocephaliformes	pelagic	40	45	15	unclear	Fig. S1 only
Maulisia argipalla	Platytroctidae	Alepocephaliformes	pelagic	40	45	15	unclear	Fig. S1 only
Mirorictus taningi	Platytroctidae	Alepocephaliformes	pelagic	40	45	15	unclear	Fig. S1 only
Sagamichthys abei	Platytroctidae	Alepocephaliformes	pelagic	40	45	15	unclear	Fig. S1 only
Thalassenchelys coheni	Colocongridae	Anguilliformes	pelagic	44	54	2	unclear	Fig. S1 only
Avocettina infans	Nemichthyidae	Anguilliformes	pelagic	27	63	10	unclear	Fig. S1 only
Nemichthys scolopaceus	Nemichthyidae	Anguilliformes	pelagic	27	63	10	unclear	Fig. S1 only
Serrivomer jesperseni	Serrivomeridae	Anguilliformes	pelagic	27	63	10	unclear	Fig. S1 only
Serrivomer sector	Serrivomeridae	Anguilliformes	pelagic	27	63	10	unclear	Fig. S1 only
Bathylagus pacificus	Microstomatidae	Argentiniformes	pelagic	58	39	3	unclear	Fig. S1 only
Lipolagus ochotensis	Microstomatidae	Argentiniformes	pelagic	58	39	3	unclear	Fig. S1 only

Pseudobathylagus milleri	Microstomatidae	Argentiniformes	pelagic	58	39	3	unclear	Fig. S1 only
Macropinna microstoma	Opisthoproctidae	Argentiniformes	pelagic	58	39	3	unclear	Fig. S1 only
Rhynchohyalus natalensis	Opisthoproctidae	Argentiniformes	pelagic	58	39	3	unclear	Fig. S1 only
Alepisaurus ferox	Alepisauridae	Aulopiformes	pelagic	6	91	3	S	Fig. S1 only
Gigantura indica	Giganturidae	Aulopiformes	pelagic	1	94	5	S	Fig. S1 only
Scopelosaurus harryi	Notosudidae	Aulopiformes	pelagic	42	54	4	unclear	Fig. S1 only
Anotopterus nikparini	Paralepididae	Aulopiformes	pelagic	6	91	3	S	Fig. S1 only
Lestidiops pacificus	Paralepididae	Aulopiformes	pelagic	6	91	3	S	Fig. S1 only
Lestidiops ringens	Paralepididae	Aulopiformes	pelagic	6	91	3	S	Fig. S1 only
Macroparalepis johnfitchi	Paralepididae	Aulopiformes	pelagic	6	91	3	S	Fig. S1 only
Magnisudis atlantica	Paralepididae	Aulopiformes	pelagic	6	91	3	S	Fig. S1 only
Sudis atrox	Paralepididae	Aulopiformes	pelagic	6	91	3	S	Fig. S1 only
Benthalbella dentata	Scopelarchidae	Aulopiformes	pelagic	39	53	8	unclear	Fig. S1 only
Benthalbella linguidens	Scopelarchidae	Aulopiformes	pelagic	39	53	8	unclear	Fig. S1 only
Barbourisia rufa	Barbourisiidae	Beryciformes	both demersal and pelagic	51	45	4	unclear	Fig. S1 only
Cetostoma regani	Cetomimidae	Beryciformes	pelagic	51	45	4	unclear	Fig. S1 only
Melamphaes lugubris	Melamphaidae	Beryciformes	pelagic	31	67	2	unclear	Fig. S1 only
Poromitra crassiceps	Melamphaidae	Beryciformes	pelagic	50	45	5	unclear	Fig. S1 only
Scopeloberyx robustus	Melamphaidae	Beryciformes	pelagic	51	44	5	unclear	Fig. S1 only

Scopelogadus mizolepis bispinosus	Melamphaidae	Beryciformes	pelagic	50	47	3	unclear	Fig. S1 only
Rondeletia loricata	Rondeletiidae	Beryciformes	pelagic	51	45	4	unclear	Fig. S1 only
Albatrossia pectoralis	Macrouridae	Gadiformes	demersal	97	0	3	N	Fig. S1 only
Coelorinchus scaphopsis	Macrouridae	Gadiformes	demersal	0	95	5	S	Fig. S1 only
Coryphaenoides armatus	Macrouridae	Gadiformes	both demersal and pelagic	60	31	9	unclear	Fig. S1 only
Coryphaenoides cinereus	Macrouridae	Gadiformes	demersal	97	0	3	N	Fig. S1 only
Coryphaenoides filifer	Macrouridae	Gadiformes	demersal	97	0	3	N	Fig. S1 only
Coryphaenoides leptolepis	Macrouridae	Gadiformes	demersal	42	55	3	unclear	Fig. S1 only
Malacocephalus laevis	Macrouridae	Gadiformes	both demersal and pelagic	0	94	6	S	Fig. S1 only
Nezumia liolepis	Macrouridae	Gadiformes	demersal	51	47	2	unclear	Fig. S1 only
Melanonus zugmayeri	Melanonidae	Gadiformes	pelagic	44	49	7	unclear	Fig. S1 only
Halargyreus johnsonii	Moridae	Gadiformes	both demersal and pelagic	89	4	7	N	Fig. S1 only
Stylephorus chordatus	Stylephoridae	Lampridiformes	pelagic	96	0	4	N	Fig. S1 only
Lampris guttatus	Lampridae	Lampriformes	pelagic	43	51	6	unclear	Fig. S1 only
Regalecus russelii	Regalecidae	Lampriformes	pelagic	0	95	5	S	Fig. S1 only
Cryptopsaras couesii	Ceratiidae	Lophiiformes	pelagic	13	83	4	S	Fig. S1 only
Chaunacops coloratus	Chaunacidae	Lophiiformes	demersal	5	91	4	S	Fig. S1 only
Gigantactis vanhoeffeni	Gigantactinidae	Lophiiformes	pelagic	13	83	4	S	Fig. S1 only
Melanocetus johnsonii	Melanocetidae	Lophiiformes	pelagic	13	83	4	S	Fig. S1 only

Bertella idiomorpha	Oneirodidae	Lophiiformes	pelagic	13	83	4	S	Fig. S1 only
Oneirodes acanthias	Oneirodidae	Lophiiformes	pelagic	38	55	7	unclear	Fig. S1 only
Oneirodes thompsoni	Oneirodidae	Lophiiformes	pelagic	41	52	7	unclear	Fig. S1 only
Benthosema panamense	Myctophidae	Myctophiformes	pelagic	0	96	4	S	Fig. S1 only
Ceratoscopelus townsendi	Myctophidae	Myctophiformes	pelagic	52	45	3	unclear	Fig. S1 only
Diaphus anderseni	Myctophidae	Myctophiformes	pelagic	0	98	2	S	Fig. S1 only
Diaphus theta	Myctophidae	Myctophiformes	pelagic	52	45	3	unclear	Fig. S1 only
Diogenichthys laternatus	Myctophidae	Myctophiformes	pelagic	0	96	4	S	Fig. S1 only
Electrona risso	Myctophidae	Myctophiformes	pelagic	45	49	6	unclear	Fig. S1 only
Gonichthys tenuiculus	Myctophidae	Myctophiformes	pelagic	0	94	6	S	Fig. S1 only
Hygophum proximum	Myctophidae	Myctophiformes	pelagic	0	96	4	S	Fig. S1 only
Hygophum reinhardtii	Myctophidae	Myctophiformes	pelagic	0	96	4	S	Fig. S1 only
Lampadena urophaos urophaos	Myctophidae	Myctophiformes	pelagic	52	45	3	unclear	Fig. S1 only
Lampanyctus tenuiformis	Myctophidae	Myctophiformes	pelagic	2	87	11	S	Fig. S1 only
Myctophum nitidulum	Myctophidae	Myctophiformes	pelagic	4	92	4	S	Fig. S1 only
Nannobrachium regale	Myctophidae	Myctophiformes	pelagic	2	87	11	S	Fig. S1 only
Nannobrachium ritteri	Myctophidae	Myctophiformes	pelagic	2	87	11	S	Fig. S1 only
Notoscopelus resplendens	Myctophidae	Myctophiformes	pelagic	4	93	3	S	Fig. S1 only
Parvilux ingens	Myctophidae	Myctophiformes	pelagic	52	45	3	unclear	Fig. S1 only

Protomyctophum crockeri	Myctophidae	Myctophiformes	pelagic	87	3	10	N	Fig. S1 only
Protomyctophum thompsoni	Myctophidae	Myctophiformes	pelagic	87	3	10	N	Fig. S1 only
Stenobrachius leucopsarus	Myctophidae	Myctophiformes	pelagic	52	45	3	unclear	Fig. S1 only
Stenobrachius nannochir	Myctophidae	Myctophiformes	pelagic	52	45	3	unclear	Fig. S1 only
Symbolophorus californiensis	Myctophidae	Myctophiformes	pelagic	0	96	4	S	Fig. S1 only
Symbolophorus evermanni	Myctophidae	Myctophiformes	pelagic	0	96	4	S	Fig. S1 only
Taaningichthys bathyphilus	Myctophidae	Myctophiformes	pelagic	52	45	3	unclear	Fig. S1 only
Tarletonbeania crenularis	Myctophidae	Myctophiformes	pelagic	7	89	4	S	Fig. S1 only
Triphoturus mexicanus	Myctophidae	Myctophiformes	pelagic	52	45	3	unclear	Fig. S1 only
Neoscopelus macrolepidotus	Neoscopelidae	Myctophiformes	demersal	69	26	5	unclear	Fig. S1 only
Scopelengys tristis	Neoscopelidae	Myctophiformes	pelagic	61	37	2	unclear	Fig. S1 only
Cataetyx rubrirostris	Bythitidae	Ophidiiformes	demersal	50	41	9	unclear	Fig. S1 only
Lamprogrammus niger	Ophidiidae	Ophidiiformes	both demersal and pelagic	39	56	5	unclear	Fig. S1 only
Acantholiparis opercularis	Liparidae	Perciformes/Cottoidei	demersal	96	1	3	N	Fig. S1 only
Careproctus colletti	Liparidae	Perciformes/Cottoidei	demersal	96	1	3	N	Fig. S1 only
Careproctus cypselurus	Liparidae	Perciformes/Cottoidei	demersal	96	1	3	N	Fig. S1 only
Careproctus gilberti	Liparidae	Perciformes/Cottoidei	demersal	96	1	3	N	Fig. S1 only
Elassodiscus caudatus	Liparidae	Perciformes/Cottoidei	demersal	96	1	3	N	Fig. S1 only
Nectoliparis pelagicus	Liparidae	Perciformes/Cottoidei	pelagic	96	1	3	N	Fig. S1 only

Paraliparis cephalus	Liparidae	Perciformes/Cottoidei	demersal	95	2	3	N	Fig. S1 only
Paraliparis dactylosus	Liparidae	Perciformes/Cottoidei	demersal	96	1	3	N	Fig. S1 only
Paraliparis pectoralis	Liparidae	Perciformes/Cottoidei	demersal	96	1	3	N	Fig. S1 only
Paraliparis rosaceus	Liparidae	Perciformes/Cottoidei	demersal	96	1	3	N	Fig. S1 only
Rhinoliparis attenuatus	Liparidae	Perciformes/Cottoidei	pelagic	96	1	3	N	Fig. S1 only
Rhinoliparis barbulifer	Liparidae	Perciformes/Cottoidei	demersal	96	1	3	N	Fig. S1 only
Psychrolutes phrictus	Psychrolutidae	Perciformes/Cottoidei	demersal	100	0	0	N	Fig. S1 only
Lycenchelys camchatica	Zoarcidae	Perciformes/Zoarcoidei	demersal	73	0	27	N	Fig. S1 only
Lycodapus endemoscotus	Zoarcidae	Perciformes/Zoarcoidei	pelagic	73	0	27	N	Fig. S1 only
Lycodapus pachysoma	Zoarcidae	Perciformes/Zoarcoidei	pelagic	73	0	27	N	Fig. S1 only
Pachycara gymninium	Zoarcidae	Perciformes/Zoarcoidei	demersal	73	0	27	N	Fig. S1 only
Pachycara lepinium	Zoarcidae	Perciformes/Zoarcoidei	demersal	73	0	27	N	Fig. S1 only
Atheresthes evermanni	Pleuronectidae	Pleuronectiformes	demersal	55	41	4	unclear	Fig. S1 only
Clidoderma asperrimum	Pleuronectidae	Pleuronectiformes	demersal	50	46	4	unclear	Fig. S1 only
Embassichthys bathybius	Pleuronectidae	Pleuronectiformes	demersal	50	46	4	unclear	Fig. S1 only
Eurypharynx pelecanoides	Eurypharyngidae	Saccopharyngiformes	pelagic	50	45	5	unclear	Fig. S1 only
Taractes asper	Bramidae	Scombriformes	pelagic	12	84	4	S	Fig. S1 only
Chiasmodon niger	Chiasmodontidae	Scombriformes	pelagic	41	52	7	unclear	Fig. S1 only
Kali indica	Chiasmodontidae	Scombriformes	pelagic	6	83	11	S	Fig. S1 only

Diplospinus multistriatus	Gempylidae	Scombriformes	pelagic	0	92	8	S	Fig. S1 only
Cubiceps baxteri	Nomeidae	Scombriformes	pelagic	0	94	6	S	Fig. S1 only
Cubiceps capensis	Nomeidae	Scombriformes	pelagic	0	97	3	S	Fig. S1 only
Psenes pellucidus	Nomeidae	Scombriformes	pelagic	0	97	3	S	Fig. S1 only
Aphanopus arigato	Trichiuridae	Scombriformes	demersal	51	40	9	unclear	Fig. S1 only
Chauliodus macouni	Chauliodontidae	Stomiiformes	pelagic	67	31	2	unclear	Fig. S1 only
Cyclothone acclinidens	Gonostomatidae	Stomiiformes	pelagic	8	87	5	S	Fig. S1 only
Cyclothone alba	Gonostomatidae	Stomiiformes	pelagic	8	87	5	S	Fig. S1 only
Cyclothone atraria	Gonostomatidae	Stomiiformes	pelagic	8	87	5	S	Fig. S1 only
Cyclothone microdon	Gonostomatidae	Stomiiformes	pelagic	8	87	5	S	Fig. S1 only
Cyclothone pallida	Gonostomatidae	Stomiiformes	pelagic	8	87	5	S	Fig. S1 only
Cyclothone pseudopallida	Gonostomatidae	Stomiiformes	pelagic	8	87	5	S	Fig. S1 only
Cyclothone signata	Gonostomatidae	Stomiiformes	pelagic	8	87	5	S	Fig. S1 only
Diplophos taenia	Gonostomatidae	Stomiiformes	pelagic	5	90	5	S	Fig. S1 only
Gonostoma atlanticum	Gonostomatidae	Stomiiformes	pelagic	8	87	5	S	Fig. S1 only
Sigmops gracilis	Gonostomatidae	Stomiiformes	pelagic	8	87	5	S	Fig. S1 only
Idiacanthus antrostomus	Idiacanthidae	Stomiiformes	pelagic	36	60	4	unclear	Fig. S1 only
Aristostomias scintillans	Malacosteidae	Stomiiformes	pelagic	41	54	5	unclear	Fig. S1 only
Photonectes margarita	Melanostomiidae	Stomiiformes	pelagic	13	84	3	S	Fig. S1 only

Tactostoma macropus	Melanostomiidae	Stomiiformes	pelagic	37	59	4	unclear	Fig. S1 only
Vinciguerria nimbaria	Phosichthyidae	Stomiiformes	pelagic	7	88	5	S	Fig. S1 only
Argyropelecus aculeatus	Sternoptychidae	Stomiiformes	pelagic	31	65	4	unclear	Fig. S1 only
Argyropelecus affinis	Sternoptychidae	Stomiiformes	pelagic	31	65	4	unclear	Fig. S1 only
Argyropelecus hemigymnus	Sternoptychidae	Stomiiformes	pelagic	31	65	4	unclear	Fig. S1 only
Argyropelecus lychnus	Sternoptychidae	Stomiiformes	pelagic	31	65	4	unclear	Fig. S1 only
Argyropelecus sladeni	Sternoptychidae	Stomiiformes	pelagic	31	65	4	unclear	Fig. S1 only
Danaphos oculatus	Sternoptychidae	Stomiiformes	pelagic	9	91	0	S	Fig. S1 only
Sternoptyx diaphana	Sternoptychidae	Stomiiformes	pelagic	7	88	5	S	Fig. S1 only
Sternoptyx obscura	Sternoptychidae	Stomiiformes	pelagic	7	88	5	S	Fig. S1 only
Sternoptyx pseudobscura	Sternoptychidae	Stomiiformes	pelagic	7	88	5	S	Fig. S1 only
Borostomias panamensis	Stomiidae	Stomiiformes	pelagic	52	44	4	unclear	Fig. S1 only
Chauliodus sloani	Stomiidae	Stomiiformes	pelagic	73	25	2	N	Fig. S1 only
Flagellostomias boureei	Stomiidae	Stomiiformes	pelagic	0	96	4	S	Fig. S1 only
Idiacanthus fasciola	Stomiidae	Stomiiformes	pelagic	36	60	4	unclear	Fig. S1 only
Malacosteus niger	Stomiidae	Stomiiformes	pelagic	41	54	5	unclear	Fig. S1 only
Neonesthes capensis	Stomiidae	Stomiiformes	pelagic	18	22	60	unclear	Fig. S1 only
Pachystomias microdon	Stomiidae	Stomiiformes	pelagic	41	54	5	unclear	Fig. S1 only
Rhadinesthes decimus	Stomiidae	Stomiiformes	pelagic	45	49	6	unclear	Fig. S1 only

Stomias atriventer	Stomiidae	Stomiiformes	pelagic	41	52	7	unclear	Fig. S1 only
Anoplogaster cornuta	Anoplogastridae	Trachichthyiformes	pelagic	53	43	4	unclear	Fig. S1 only
Diretmus argenteus	Diretmidae	Trachichthyiformes	pelagic	0	91	9	S	Fig. S1 only

Table S5: Region-of-origin for California cartilaginous fishes as inferred from BioGeoBEARS analyses. Blue rows= southern origin species; orange rows= northern origin species; unshaded rows= species with unclear origin.

species	order	family	habitat	# maps inferring northern origin	# maps inferring southern origin	# maps indet.	region of origin coded in plots	notes
Carcharhinus brachyurus	Carcharhiniformes	Carcharhinidae	pelagic	0	95	5	S	
Carcharhinus leucas	Carcharhiniformes	Carcharhinidae	pelagic	0	96	4	S	
Carcharhinus obscurus	Carcharhiniformes	Carcharhinidae	pelagic	0	98	2	S	
Nasolamia velox	Carcharhiniformes	Carcharhinidae	demersal	0	95	5	S	
Prionace glauca	Carcharhiniformes	Carcharhinidae	pelagic	1	97	2	S	
Rhizoprionodon longurio	Carcharhiniformes	Carcharhinidae	demersal	0	94	6	S	
Carcharodon carcharias	Carcharhiniformes	Lamnidae	pelagic	10	88	2	S	
Apristurus brunneus	Carcharhiniformes	Pentanchidae	both demersal and pelagic	44	50	6	unclear	
Apristurus kampae	Carcharhiniformes	Pentanchidae	demersal	44	53	3	unclear	
Parmaturus xaniurus	Carcharhiniformes	Pentanchidae	demersal	53	41	6	unclear	
Cephaloscyllium ventriosum	Carcharhiniformes	Scyliorhinidae	demersal	0	94	6	S	
Sphyrna lewini	Carcharhiniformes	Sphyrnidae	pelagic	0	99	1	S	
Galeorhinus galeus	Carcharhiniformes	Triakidae	pelagic	43	49	8	unclear	
Mustelus californicus	Carcharhiniformes	Triakidae	demersal	0	97	3	S	
Mustelus henlei	Carcharhiniformes	Triakidae	demersal	42	45	13	unclear	
Mustelus lunulatus	Carcharhiniformes	Triakidae	demersal	0	96	4	S	
Triakis semifasciata	Carcharhiniformes	Triakidae	demersal	32	42	26	unclear	
Hydrolagus colliei	Chimaeriformes	Chimaeridae	demersal	51	40	9	unclear	
Hydrolagus melanophasma	Chimaeriformes	Chimaeridae	demersal	1	92	7	S	
Echinorhinus cookei	Echinorhiniformes	Echinorhinidae	demersal	49	46	5	unclear	
Heterodontus francisci	Heterodontiformes	Heterodontidae	demersal	1	96	3	S	
Chlamydoselachus anguineus	Hexanchiformes	Chlamydoselachidae	demersal	28	69	3	unclear	
Hexanchus griseus	Hexanchiformes	Hexanchidae	demersal	28	69	3	unclear	
Notorynchus cepedianus	Hexanchiformes	Hexanchidae	demersal	28	69	3	unclear	
Alopias superciliosus	Lamniformes	Alopiidae	pelagic	10	88	2	S	
Alopias vulpinus	Lamniformes	Alopiidae	pelagic	10	88	2	S	

Cetorhinus maximus	Lamniformes	Cetorhinidae	pelagic	10	88	2	S	
Isurus oxyrinchus	Lamniformes	Lamnidae	pelagic	10	88	2	S	
Lamna ditropis	Lamniformes	Lamnidae	pelagic	10	88	2	S	
Dasyatis dipterura	Myliobatiformes	Dasyatidae	demersal	0	91	9	S	
Pteroplatytrygon violacea	Myliobatiformes	Dasyatidae	pelagic	41	43	16	unclear	
Gymnura marmorata	Myliobatiformes	Gymnuridae	demersal	0	93	7	S	
Manta birostris	Myliobatiformes	Mobulidae	pelagic	0	100	0	S	
Myliobatis californicus	Myliobatiformes	Myliobatidae	demersal	38	53	9	unclear	
Urobatis halleri	Myliobatiformes	Urotrygonidae	demersal	0	100	0	S	
Rhincodon typus	Orectolobiformes	Rhincodontidae	pelagic	1	87	12	S	
Bathyraja abyssicola	Rajiformes	Arhynchobatidae	demersal	98	1	1	N	
Bathyraja aleutica	Rajiformes	Arhynchobatidae	demersal	98	1	1	N	
Amblyraja hyperborea	Rajiformes	Rajidae	demersal	53	44	3	unclear	
Beringraja binoculata	Rajiformes	Rajidae	demersal	52	44	4	unclear	
Raja inornata	Rajiformes	Rajidae	demersal	52	44	4	unclear	
Raja rhina	Rajiformes	Rajidae	demersal	52	44	4	unclear	
Raja stellulata	Rajiformes	Rajidae	demersal	52	44	4	unclear	
Rhinobatos productus	Rhinopristiformes	Rhinobatidae	demersal	0	98	2	S	
Zapteryx exasperata	Rhinopristiformes	Trygonorrhinidae	demersal	1	94	5	S	
Euprotomicrus bispinatus	Squaliformes	Dalatiidae	pelagic	0	99	1	S	
Isistius brasiliensis	Squaliformes	Dalatiidae	pelagic	0	97	3	S	
Somniosus pacificus	Squaliformes	Somniosidae	demersal	47	48	5	unclear	
Zameus squamulosus	Squaliformes	Somniosidae	demersal	0	95	5	S	
Squalus acanthias	Squaliformes	Squalidae	demersal	40	28	32	unclear	
Squatina californica	Squatiniformes	Squatinidae	demersal	43	53	4	unclear	
Platyrhinoidis triseriata	Torpediniformes	Platyrhinidae	demersal	0	96	4	S	
Torpedo californica	Torpediniformes	Torpedinidae	demersal	47	48	5	unclear	
Harriotta raleighana	Chimaeriformes	Rhinochimaeridae	demersal	0	91	9	S	Fig. S1 only
